# Fit-for-Purpose: Species Distribution Model Performance Depends on Evaluation Criteria – Dutch Hoverflies as a Case Study

**DOI:** 10.1371/journal.pone.0063708

**Published:** 2013-05-14

**Authors:** Jesús Aguirre-Gutiérrez, Luísa G. Carvalheiro, Chiara Polce, E. Emiel van Loon, Niels Raes, Menno Reemer, Jacobus C. Biesmeijer

**Affiliations:** 1 Naturalis Biodiversity Center, Leiden, The Netherlands; 2 Institute for Biodiversity and Ecosystems Dynamics (IBED), University of Amsterdam, Amsterdam, The Netherlands; 3 Institute of Integrative and Comparative Biology, University of Leeds, Leeds, United Kingdom; 4 Leiden University, Section National Herbarium of the Netherlands, Leiden, The Netherlands; 5 European Invertebrate Survey – The Netherlands, Leiden, The Netherlands; University of Sydney, Australia

## Abstract

Understanding species distributions and the factors limiting them is an important topic in ecology and conservation, including in nature reserve selection and predicting climate change impacts. While Species Distribution Models (SDM) are the main tool used for these purposes, choosing the best SDM algorithm is not straightforward as these are plentiful and can be applied in many different ways. SDM are used mainly to gain insight in 1) overall species distributions, 2) their past-present-future probability of occurrence and/or 3) to understand their ecological niche limits (also referred to as ecological niche modelling). The fact that these three aims may require different models and outputs is, however, rarely considered and has not been evaluated consistently. Here we use data from a systematically sampled set of species occurrences to specifically test the performance of Species Distribution Models across several commonly used algorithms. Species range in distribution patterns from rare to common and from local to widespread. We compare overall model fit (representing species distribution), the accuracy of the predictions at multiple spatial scales, and the consistency in selection of environmental correlations all across multiple modelling runs. As expected, the choice of modelling algorithm determines model outcome. However, model quality depends not only on the algorithm, but also on the measure of model fit used and the scale at which it is used. Although model fit was higher for the consensus approach and Maxent, Maxent and GAM models were more consistent in estimating local occurrence, while RF and GBM showed higher consistency in environmental variables selection. Model outcomes diverged more for narrowly distributed species than for widespread species. We suggest that matching study aims with modelling approach is essential in Species Distribution Models, and provide suggestions how to do this for different modelling aims and species’ data characteristics (*i.e.* sample size, spatial distribution).

## Introduction

Understanding current and predicting future distributions of species is pivotal for ecology and for implementation of biodiversity conservation and policy measures (e.g. International Union for Conservation of Nature -IUCN Red Lists; reserve selection). One of the most common methods used to gain insight in species distributions and environmental niches is Species Distribution Modelling [1], which is also referred to as ecological niche modelling (see discussions on terminology in [2], [3], [4], [5], [6]). SDM identifies locations with suitable (a)biotic conditions for species occurrences, based on climatological, environmental and/or biotic correlates [7]. A broad range of algorithms [8], [9] and platforms (*i.e*. BIOMOD, ModEco, OpenModeller, [10]–[12]) can be used to fit the models, each with unique features, such as different variable selecting techniques or methods for selecting (pseudo) absences [13]–[16]. Consequently, the best fitted model depends not only on presence data available, but also strongly on the modelling approach [17], [18]. SDMs are used mainly to (1) gain insight in species’ overall distribution (*i.e.* [19], [20]), (2) obtain predicted occurrences for specific locations (*i.e.* [21], [22]) or (3) understand niche limits of species (*i.e.* [4], [23]–[25]). Several studies point to the need to evaluate and validate SDMs and perform in-depth analyses of the impact of algorithm selection and within algorithm consistency of predictions to generate more meaningful models [2], [26]. For example, using virtual species, Saupe et al. [25] found that the distribution of the species data used for model training with regard to the environmental conditions available influences modelling results. Wisz et al. [27] showed that model accuracy (AUC values) depends on the algorithm used, reinforcing the need to assess performance of different modelling techniques [28], including consensus methods (that integrate the predictions of several algorithms) [29]. Lastly, Zimmermann et al. [30] showed how SDM can be tailored to satisfy different aims and improve prediction accuracy. However, our screening of recent papers using SDM (see Table S1 in Supplementary material) shows that studies modelling a single species tend to use one algorithm, whereas studies modelling multiple species tend to use multiple algorithms, generally without clear explanation of the reasons for algorithms selection criteria. The 19 algorithms used in a set of 42 recent papers (Table S1) occur in both, single and multi-species studies, with Maxent (Maximum entropy) and GLM (Generalized Linear Models) being two of the most common ones. However, none of these studies analyse the advantages/disadvantages of selecting one or more algorithms, being still unclear whether species-specific features such as level of rarity, geographic spread or a combination of both, affect model fit (but see Table S1).

Here we investigate which species distribution modelling algorithms perform most consistently when: (1) evaluating overall model fit; (2) evaluating spatial predictions of species occurrence at patch, landscape and regional scales; and (3) identifying environmental factors as important correlates of species occurrence. We test these three aspects for a group of well-sampled hoverfly species in the Netherlands, that are selected such that they include rare to common and local to widespread species.

## Methods

### Species Data and Selection

We used presence-only records for Dutch hoverflies (Diptera: Syrphidae) in the Netherlands from the European Invertebrate Survey [31] collected during the last ten years (2000–2010). This database contains more than 400, 000 records of 328 species over a time span of 200 years for the entire country (Fig. S1). For the species selection we first characterised all species in terms of occupancy (rare to common, based on the number of 1 km^2^ cells occupied) and spatial distribution (narrowly distributed to widespread). Spatial distribution measure was calculated as the longest distance found within the 3^th^ quartile of distances between all recorded locations for that species. We chose the 3^th^ quartile distance as it may better represent the records distribution in geographic space, avoiding any outlier present in the last quartile. We then extracted a total of 16 species that clearly belonged to one of the following four groups: narrowly distributed and rare (NR), narrowly distributed and common (NC), widely distributed and rare (WR), and widely distributed and common (WC). The selected species ranged in number of records from 6 to 2094 and in spatial distribution from 3.2 to 238.4 Km 3rd quartile distance (Table S2).

### Environmental Data used for Modelling

We obtained bioclimatic data from WorldClim [32], as its variables render biologically meaningful estimates representing annual trends in seasonality and extreme/limiting factors. We did not include additional environmental variables, as our objective was not an in-depth study of the ecology of the hoverfly species but rather of the consistency of performance of the different algorithms. The selected species covered most of the range in environmental space of the Netherlands (Fig. S2). To reduce co-linearity between predictors [1], we only retained variables with a Pearson’s pair-wise correlation coefficient <|0.7|. When two variables were highly correlated we chose the one least correlated to others, leading to a total of nine climatic and one topographic variables with a spatial resolution of 1 km^2^ selected for the construction of the species distribution models (Table S3).

### Modelling Algorithms

We fitted species distribution models (SDM) using six commonly used algorithms (see Table S1): four machine learning methods, Artificial Neural Networks (ANN, [33]), Generalized Boosted Models (GBM, [34], [35]), Random Forests (RF, [36]) and Maximum Entropy modelling (Maxent, [37]); and two regression methods, Generalized Additive Models (GAM, [38]), Generalized Linear Models (GLM, [39]). We did not use “true absence” data, using instead a random or a given sample of background points as pseudo-absences. These algorithms have been applied for modelling environmental relationships for a wide range of species [8]–[10], [13], [27], [37], [40]. We used the BIOMOD package [10] (v. 1.1–7.00) for R [41] for all selected algorithms, except Maxent, for which we used the Maximum Entropy Modelling software MaxEnt (v3.3.3e,www.cs.princeton.edu/~schapire/maxent/). We followed default settings recommended by Thuiller et al. [42] (for BIOMOD) and Phillips and Dudik [43] (MaxEnt) for fitting the models. As every run within the ANN algorithm can render different results we selected the best weight decay and the number of units in the hidden layer by carrying out five-fold cross-validation runs. The GAM models were run with a spline function with three degrees of smoothing. The GBM models were constructed by fitting 5000 trees and five cross-validations in order to identify the number of trees that produced most accurate predictions. GLM’s were generated by using polynomial terms with the stepwise procedure and using the Akaike Information Criterion (AIC) for model selection. For RF models 500 trees were used as the building criterion following other studies that have implemented the algorithm successfully with these settings (see [2], [44]–[46]). MaxEnt was run with the auto-features option and the logistic output format was used as this has proven to be the appropriate method in an extensive multispecies study carried out by Phillips and Dudik [43]. Finally, a consensus ensemble approach [47], hereafter “Consensus approach”, was applied using the BIOMOD platform models generated by GLM, GAM, GBM, RF and ANN. The Consensus approach is thought to offer more robust predictions for the potential and realized distribution of species than single algorithms [47]. Maxent is not integrated in BIOMOD v1.1–7.00, and therefore it was not part of the Consensus approach. The Consensus approach implementation consisted of the ensemble of the 10 model repetitions×5 modelling algorithms = 50 output maps. We used the Receiving Operating Characteristic (ROC) of the area under de curve mean method [48] to create consensus maps [10]. In this method, after converting the outputs to binary predictions using their correspondent thresholds that maximize the sensitivity and specificity of the models [49], every cell for which more than half of the models predicted a presence, was considered a presence, the other cells were assigned as absence. All single modelling algorithms were run for the 16 hoverfly species. For each species and algorithm ten replicate runs were applied (two species had only 6 and 8 number of occurrence records and for these respectively 6 and 8 replicate runs were conducted).

### Modelling Process

To generate the species distribution models, all modelling algorithms used in this study required the input of (pseudo) absences (BIOMOD) or background points (MaxEnt) [40], [50], [51]. Pseudo-absences were randomly selected locations where the focal species was not present but other hoverfly species had been found (more than 9000 Km^2^ cells conforming the total species modelled and available for generation of pseudo absences). This approach is more objective and realistic than taking pseudo-absences from sites that have not been sampled at all, accounting for the possible sampling bias [52], [53], and likely providing more accurate results [40], [50]. For every species, the presence records were randomly partitioned in 75% for training and 25% for testing and were the same for all algorithms but Maxent, which was run in a separated platform. This random partitioning was repeated ten times to obtain a robust estimate for the algorithm’s performance [8]. We generated and compared a total of 1078 models for the 16 selected species (16 species×7 algorithms (incl. consensus)×6–10 cross-validation runs).

### Evaluation of Results Across Modelling Algorithms

Comparing the quality and accuracy of SDMs is generally achieved by comparing prediction success, however, this represents a limited view of the models accuracy [54]. Therefore, we evaluate the SDMs in three different ways: a) comparing the Area Under the Curve (AUC) values to assess differences in the general model fit, b) comparing the geographical consistency of the maps produced by each of the algorithms to assess the spatial congruence in presence and absence predictions; and c) comparing the contribution of the various environmental variables to the different models to assess the consistency of variable selection and contribution between runs within algorithm. Together these assessments provide a more robust and better evaluation of the performance of the different algorithms and insight into general model fit (a), spatial congruence of the maps (b) and the species’ niche characterisation (c).

#### Comparing model fit across algorithms: AUC

To obtain a measure of the accuracy of the constructed SDMs the AUC of the ROC has been used. This measure is not only threshold independent but also evaluates both the false-positive error rate and the true positive rate in order to obtain a measure for the accuracy of the constructed model. AUC values range from 0 to 1, with values below 0.5 representing a model that is not better than random and values of 1 represent models that are highly accurate [44]. For our AUC evaluations, we obtained the AUC values from each of the models created by the 10 repetitions for each species and per algorithm, including the consensus approach. Although this metric has been highly criticized in some recent studies [21], [55], it is still the most applied measure of accuracy for SDMs and that is why we considered it for our analysis. Moreover, one of the aims of this paper is to show that other accuracy measures, such as consistency of spatial predictions and of environmental variables selection may render different results compared to AUC.

#### Geographical consistency of predicted distributions

Species occurrence maps are the end product of most SDM. However, models with similar AUC values do not necessarily predict occurrences in the same locations. To assess how consistent the spatially explicit predictions of presence and absence are within and between algorithms, we calculated the similarity of the maps produced in replicate runs and compared similarity across algorithms. The SDM map similarity was assessed by creating the binary predictions (presence/absence maps) for each run using the threshold that minimizes the difference between sensitivity and specificity for each of the models [49]. Next, the 10 presence-absence maps were compared pair-wise (45 comparisons) to obtain map similarity values per algorithm per species.

Spatial accuracy can be evaluated at different scales [56], [57]. Analysing patterns at different spatial scales is a common procedure, *i.e.* the ecological neighbour theory of Addicott et al. [58] or the work of Wiens [59], and relevant to identify the ecological process and spatial needs of the species. For example, the relationship between plant diversity and ecosystem functioning was found to be scale dependent [60], [61].

We apply three different statistics incorporated in the Map Comparison Kit [62] to assess geographical patterns at different scales from the binary SDM output maps. For evaluations at small scale (single cell: 1 km^2^) we used Cohen’s Kappa statistic [63]. For medium scale evaluations, we used the Improved Fuzzy Kappa [64], which also takes values of surrounding cells into account (radius of neighbourhood of 4 cells). For large scale similarity we used the Fuzzy Global Matching [65], which evaluates overlap in patches of cells by taking into account their area of intersection, area of disagreement and the size of the patch. The latter two metrics make use of the fuzzy set theory to extract similarity values [64].

#### Consistency in environmental variables used to predict distributions

To evaluate the consistency in the strength assigned to each of the environmental variables in cross-validation SDM runs, we estimated the importance values of each variable per algorithm per species, as described by Thuiller et al. [42]. To obtain consistency values for each variable per model, species and algorithm we calculated the absolute difference between each of the importance values obtained for each of the 10 model runs and the average variable importance (average of the 10 model runs). We refer to this as the “deviance from average variable contribution”. A high deviance indicates a high variance in variable importance across runs. This analysis was not performed for the consensus approach as it is composed of all BIOMOD algorithms and a combined variable contribution value cannot be defined in a meaningful way for an ensemble model.

#### Overall analysis of results

We used Linear Mixed Effects Models (LME) [66] to investigate the possible effect of algorithm, the number of records and their spatial distribution on the attained AUC values, the geographic prediction similarity (Kappa, Improved Fuzzy Kappa and Fuzzy Global Matching) and the environmental variable contributions.

We fitted the LME in the R platform using the “nlme” package [67]. To improve the normality of the data a logit transformation was applied to the response variables AUC and Map similarity and a log transformation to the DFAC values. We used the number of records, spatial distribution of the records (upper value of 3rd quartile distance) and the algorithm as the fixed effects and the species as the random effect for the AUC and Map similarity. To account for the non-independence of the predictions generated based on the data from a given species, species identity was included as a random effect. Finally, we evaluated the consistency in variable contribution to the SDMs with a LME that included the environmental variable and algorithm as fixed effects and species as a random effect.

## Results

### Comparing Model Fit Across Algorithms: AUC

**Figure 1:**
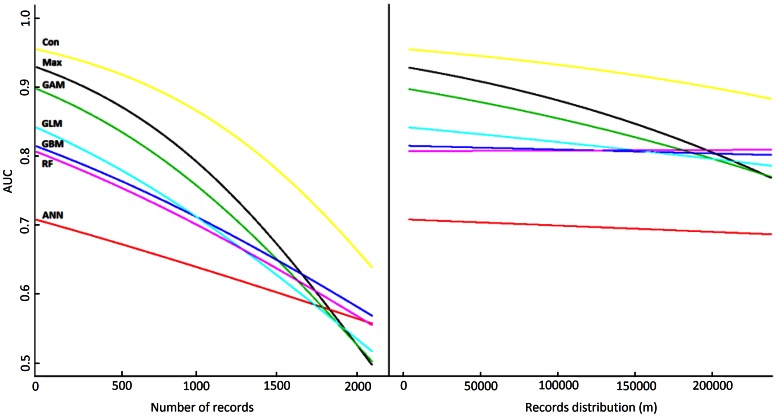
Effect of records’ availability and spatial distribution on model fit. Effect of records availability and spatial distribution on model fit based on the AUC evaluation of the different algorithms. For the AUC evaluation, we present the back-transformed mean values estimated using Linear Mixed Effect models for each algorithm. The first column presents the results with relation to the number of records and the second with relation to the records distribution.

AUC values differed significantly between algorithms (Fig. S3) and significantly declined with increasing number of records (Fig. 1). The Consensus approach obtained the highest AUC values, independently of the number of records and the spatial distribution. The next best models in terms of model fit were Maxent and GAM, which had significantly higher AUC values than GLM, GBM, RF, especially at low numbers of records, while ANN performed significantly worse (Table 1). Spatial distribution did not significantly affect model fit (only weakly for Maxent. Table 1, Table S4).

**Table 1:** Results of the Linear Mixed Effect models for the AUC, Kappa, IFK, FGM and DFAC (deviance from average variable contribution).

Algorithms	AUC	Kappa	IFK	FGM	DFAC
Max vs ANN	(+) ***	(+) ***	(+) ***	(+) ***	(−) ***
Max vs GAM	ns	ns	ns	ns	(−) ***
Max vs GBM	(+) ***	(+) ***	ns	(−) ***	(+) ***
Max vs GLM	(+) ***	(+) ***	ns	ns	(−) ***
Max vs RF	(+) ***	ns	ns	(−) ***	(+) ***
Max vs Con	(−) *	(+) ***	(+) **	ns	Na
ANN vs GAM	(−) ***	(−) ***	(−) ***	(−) ***	(+) ***
ANN vs GBM	(−) **	(−) ***	(−) ***	(−) ***	(+) ***
ANN vs GLM	(−) ***	(−) ***	(−) ***	(−) ***	ns
ANN vs RF	(−) *	(−) ***	(−) ***	(−) ***	(+) ***
ANN vs Con	(−) ***	(−) ***	(−) ***	(−) ***	na
GAM vs GBM	(+) ***	ns	ns	(−) ***	(+) ***
GAM vs GLM	(+) *	ns	ns	ns	(−) ***
GAM vs RF	(+) ***	ns	ns	(−) ***	(+) ***
GAM vs Con	(−) ***	(+) *	ns	ns	na
GBM vs GLM	ns	ns	ns	(+) ***	(−) ***
GBM vs RF	ns	ns	ns	(−) **	(+) ***
GBM vs Con	(−) ***	ns	ns	(+) ***	na
GLM vs RF	ns	ns	ns	(−) ***	(+) ***
GLM vs Con	(−) ***	ns	ns	ns	na
RF vs Con	(−) ***	(+) *	ns	(+) ***	na
Max vs Records	(−) ***	ns	ns	(−) **	na
ANN vs Records	ns	(+) *	(+) *	ns	na
GAM vs Records	(−) ***	ns	ns	(−) **	na
GBM vs Records	(−) *	ns	ns	(−) ***	na
GLM vs Records	(−) **	ns	ns	(−) **	na
RF vs Records	(−) *	ns	ns	(−) ***	na
Con vs Records	(−) ***	ns	ns	(−) ***	na
Max vs Distribution	(−) *	ns	ns	(−) *	na
ANN vs Distribution	ns	(+) ***	(+) **	(+) *	na
GAM vs Distribution	ns	ns	ns	(−) **	na
GBM vs Distribution	ns	ns	ns	ns	na
GLM vs Distribution	ns	ns	ns	(−) *	na
RF vs Distribution	ns	ns	ns	ns	na
Con vs Distribution	ns	ns	ns	ns	na

The significance of the pairwise algorithms comparisons, their interaction with the number of records and spatial distribution is presented. The positive and negative signs apply for the first algorithm being compared against the second. For the first four measures the positive sign points to algorithms that render higher values -better fits and maps similarities. In the DFAC, the negative signs point to a more consistent algorithm as it renders lower deviances than the second. Max = Maxent, Con = Consensus approach; ns = no significant; na = not applicable. Signif. codes: 0 ‘***’ 0.001 ‘**’ 0.01 ‘*’ 0.05. Corrected Tukey’s *P values* reported.

### Geographical Consistency of Predicted Distributions

**Figure 2:**
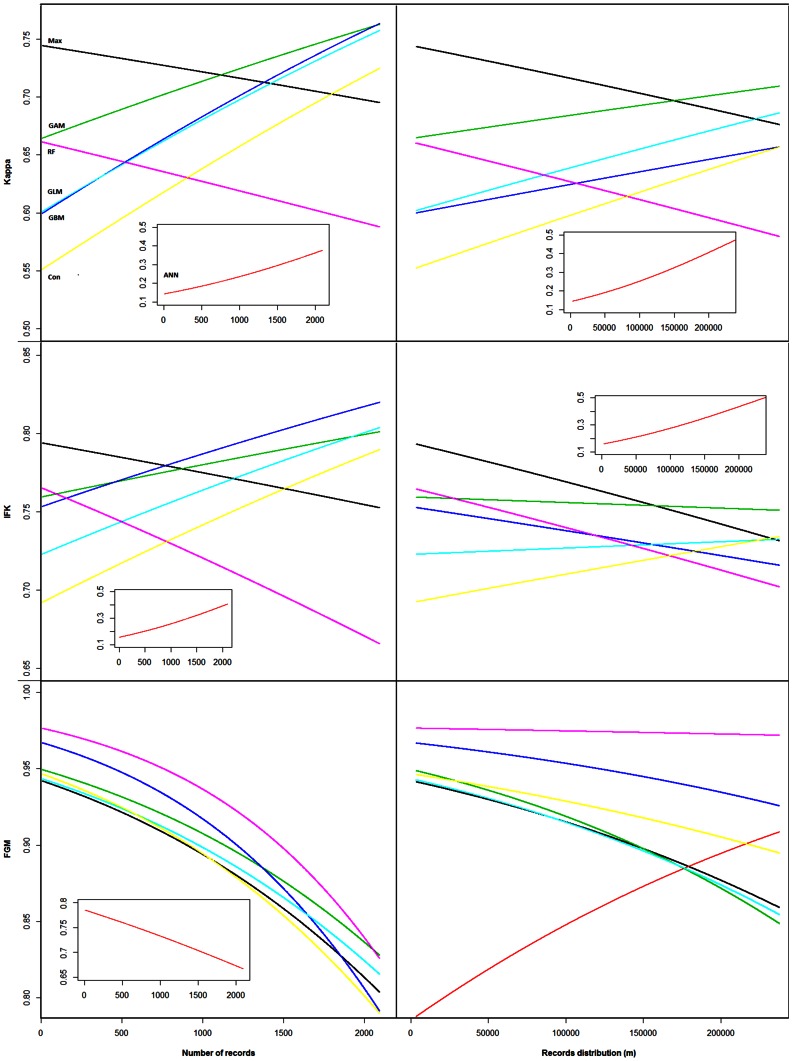
Effect of records’ availability and spatial distribution on geographical consistency. Effect of records availability and spatial distribution on geographical consistency of the different algorithms. For each spatial scale (small scale –Kappa; medium scale – IFK; and large scale - FGM), we present the back-transformed mean values estimated using Linear Mixed Effect models for each algorithm. The first column presents the results with relation with the number of records and the second with relation with the records distribution. For clarity of comparisons, ANN results are presented separately whenever its values were much lower than those obtained for other algorithms. See Tables 1 and S5 for further statistical information.

The spatial scale at which maps were compared strongly affected the spatial congruence within algorithms. At the largest scale (Fuzzy global matching comparison, “FGM”) all algorithms rendered high spatial congruence across model runs, while spatial congruence was lower at medium scale (Improved Fuzzy Kappa comparison, “IFK”) and again lower when individual (1 km2) cells were compared (Kappa comparison) (Fig. 2). This is expected, because the first two methods buffer against small mismatches between maps [64]. For all algorithms except ANN, spatial congruence was not significantly affected by number of records or spatial distribution of the data (at small and medium scales, Table 1). ANN spatial congruence improved with increasing number of records (small and medium scales) and wider distribution (all scales) of the data.

At small scale (i.e. using the Kappa statistic), Maxent and GAM produced the highest spatial consistency. RF, GBM, GLM and the Consensus approach performed similarly when number of records was high but significantly worse at low number of records (Fig. 2, Kappa panel). ANN models produced the lowest spatial consistency at both small and medium spatial scale, at the latter scale joined by a poorly performing Consensus approach. At medium spatial scale, Maxent rendered the highest spatial consistency values, but as above several other algorithms, GAM, GBM, GLM and RF, were not significantly worse (Fig. 2, IFK panel, Table 1, Table S6). GBMs and RF performed better than the other algorithms at large spatial scale (with all rendering high map similarities; Fig. 2 FGM panel, Table 1, Table S7). This improvement may, however, be due to overfitting as they mostly predict small presence patches matching closely to the locations where the training records are found (example for RF in Fig. S4).

### Environmental Consistency of Predicted Distributions

**Figure 3:**
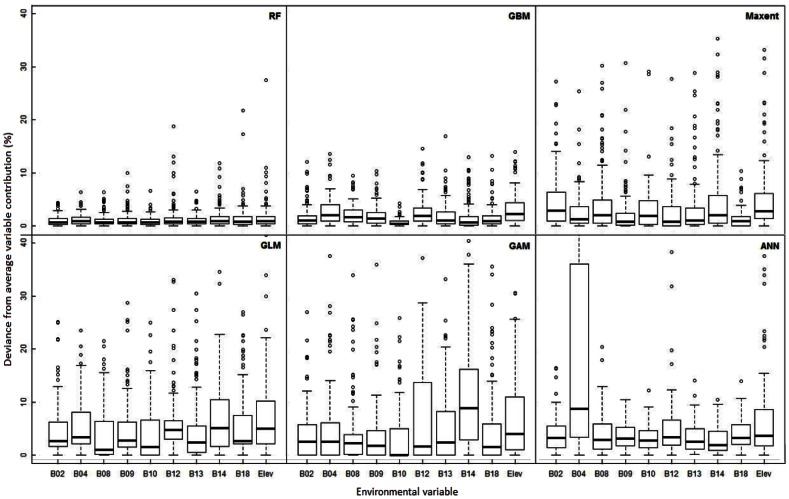
Consistency of the variables’ contribution to the model. Variability of the contribution of each environmental variable (i.e. deviance from the average variable contribution to the model) for each algorithm. In the Y axis higher deviance represents a lower consistency in the contribution values given by the algorithm to the different variables across runs. The values for variable “B04” in the ANN algorithm go to 80% and other variables present outliers going beyond the 40%, however, for plotting convenience we show only the deviance up to the 40%. See Table 1 and Tables S8 and S9 for further statistical information.

There were significant differences in how consistently algorithms assign importance to environmental variables between different runs (Table 1, Table S8). GBM and RF were the most consistent algorithms, followed by Maxent, while ANN, GAM and GLM rendered significantly higher variability across runs (Fig. 3). Variable assignment was often less consistent at small sample sizes (for ANN, GAM, GLM and RF; Fig. S5). The spatial distribution of the data affected the consistency in variable importance assignment for all algorithms for at least one variable (Fig. S6).

## Discussion

Species distribution modelling is currently the main method for predicting species distributions, which in turn may guide conservation management actions. SDM can be implemented using a range of different algorithms, whose performances are analysed in this study in three different but complementary ways, by comparing model fit, consistency of spatial predictions and consistency of the selection of environmental variables. We show that depending on the research objectives, number of records and spatial distribution of such records the most suitable algorithm will vary.

### The Model Fit

The decline of model fit (AUC) with increasing number of records is expected when using pseudo-absences or background data because the maximum attainable AUC value decreases with number of records (maximum AUC = (1-area occupied)/2) [37], [52], [68]. For comparisons of model fit between species the (bias corrected) null model approach would be more appropriate [52], but here we only compared model fit within species.

As in other studies [29], [69], the Consensus approach rendered the best overall model fit, probably because presence predictions are strictly limited to cells for which the majority of the models in the ensemble predict a presence. However, considering only AUC scores as an evaluation method for model performance may not always be the best approach [54], as AUC is not indicative of geographical and environmental consistency of a model (see below). Even though the Consensus approach produced good general fits, its drawbacks become apparent when using other performance measures (Table 2).

**Table 2:** Summary of the algorithms’ performance across analyses and the different aims for which they attain better results (for more details see Figs. 1, 2, 3).

Algorithm	Model fit-AUC values	Binary PredictionsSimilarity	Consistency in Environmental Variables selection	Observations
*Consensus* *approach*	High	Low at fine scale Medium atmedium scale Mediumat coarse scale	NA*	-Good for high model fit for narrow, wide, small and big sample sizes. It is not the best option for similarity in spatial distribution.
*Maxent*	High	High at fine scale High atmedium scale Mediumat coarse scale	Medium to high	-High scores for narrow and moderately wide distribution of records, also good for small and moderately big sample sizes (up to around 1700 records).
*GAM*	Medium	Medium at fine scaleMedium at medium scaleMedium at coarse scale	Low	-For narrow and moderately wide distribution of records, also good for small and moderately big sample sizes (around 1400 records).
*GBM*	Low	Low at fine scale Medium atmedium scale Highat coarse scale	High	-Obtains higher scores than others for common and widespread records. Obtains lower scores with small and narrow records’ distribution.
*GLM*	Low	Low at fine scale Mediumat medium scaleMedium at coarse scale	Low	-Preferred for common and widespread records although not the best in any comparison metric. Obtains lower scores with small and narrow records’ distribution.
*RF*	Low	Medium at fine scale Mediumat medium scaleHigh at coarse scale	High	-Good for common and widespread record. Obtains lower scores with small and narrow records’ distribution. Similar to GBM
*ANN*	Very low	Very low at fine scale Very lowat medium scale Verylow at coarse scale	Low	-Not better than other in most of the comparisons. It produces low scores across analysis.

* Not Available for this method.

Maxent’s better performance in comparison to the other “single” algorithms might be partly due to how the environmental variables and their interactions are modelled, i.e. incorporating progressively more mathematical complexity of the model when more data are available [37], [53]. It also seems that generative methods in general (Maxent, but also RF and GBM) render better results with small sample sizes, maybe due to faster convergence to their higher asymptotic error than discriminative methods [70]. In comparison, discriminative methods such as GLM and GAM improve their accuracy as the number of records increases and may even surpass results offered by generative methods at large sample sizes (see Fig. 1 at around 1700 records). However, for most taxa and regions, data availability rarely reaches the point where advantages of discriminative methods can be benefitted from [50], [71]. Finally, thanks to its regularization procedure, Maxent models are less likely to overfit the data [37], [53], than RF and GBM models (as shown in Fig. S4, and other recent studies, [14], [72]).

### Obtaining Geographically Consistent Predicted Distributions

Our results show that a high AUC value is not necessarily associated with a high spatial accuracy of the models (e.g. for Consensus approach in our study). However, algorithms with low AUC values produced very inconsistent spatial predictions (see Figs. 1 and 3). Moreover, the accuracy of the occurrence predictions depended on the spatial scale used. Here we used scales that roughly represent small (sub) populations (1 km^2^ cell comparison), landscape level patterns (several km^2^ area) or regional populations. If we focus on small and medium scale geographic processes, Maxent, GAM and RF models attain the best results predicting consistently the same geographic areas across repetitions (Fig. 2, Kappa and IFK panels). This result suggests that these algorithms are preferable when modelling species that are narrowly distributed and from which not many record locations are available. However, at larger spatial scales all algorithms produce highly accurate and largely similar results (with the exception of ANN), RF and GBM obtaining only slightly better results (Fig. 2, FGM panel). This suggests that when focusing on processes occurring at regional or country scale, RF and GBM algorithms might be preferable. However, due to their tendency to overfit (Fig. S4), the usefulness of these algorithms for temporal or spatial extrapolation is limited.

### How Consistent are SDM Algorithms When Selecting Significant Environmental Variables?

From the six algorithms, RF and GBM were the most consistent when selecting the environmental factors that are considered to limit the species distributions (Fig. 3). However, these algorithms tend to under-predict the species range because of overfitting the models to the training data, which is apparent by the poor predictions of the test data, as shown by the low AUC values (Fig. 1). In such cases these algorithms only detect part of the realized niche of the species and underestimate the areas that the species could potentially inhabit. Therefore, if we are only interested in the environmental niche of a species these two algorithms provide better results in our evaluation. However, there are other algorithms that performed almost as good in the consistent selection of environmental variables, while not highly overfitting the data (e.g. Maxent, see also AUC evaluation). These might be a good option for a more consistent selection of the species’ important environmental variables.

### Implications for Species Distribution Modelling

Setting the aim of the SDM exercise beforehand is key for obtaining appropriate SDMs [26]. SDM studies are performed with different main aims in mind (e.g. estimating potential general distribution, obtaining past, present or future spatial predictions, environmental niche characterization, summarized in Table 2). Our study clearly shows that depending on the objective of the study different algorithms should be selected for SDM. For example, if a conservation practitioner needs to know what the likelihood is of a species occurring in a small nature reserve then using a model with a high spatial congruence and high fit is essential. On the other hand, if one wants to understand the environmental conditions that most likely limit a species’ distribution, an algorithm with high consistency in variable strength assessment is more important. If one would be interested in a balance between the above then yet another algorithm might be preferred. In our analysis Maxent obtained some of the best results across evaluation criteria and might thus be a good starting point from among the readily available modelling options (Table 2), whereas for specific questions several other algorithms give similar quality results or might be preferred, e.g. RF for consistency in environmental variable selection.

Our results are representative of the currently implemented versions of the different algorithms and it is likely that future changes in coding the algorithms may lead to performance improvements. Moreover, while these results are only representative for the set of conditions present in the study area (The Netherlands) and caution must be taken in extrapolating our findings to areas that are substantially different, the extent and high quality of the database here used (Netherlands hoverfly database, where pseudo-absences selected for the models are likely closely related to real absences), allowed us to select the species with variable distribution patterns following objective criteria, thus making it possible to carry out algorithms comparisons with real instead of virtual data. Further work is needed to corroborate our results for areas with broader spatial and environmental range.

### Conclusion

**Figure 4:**
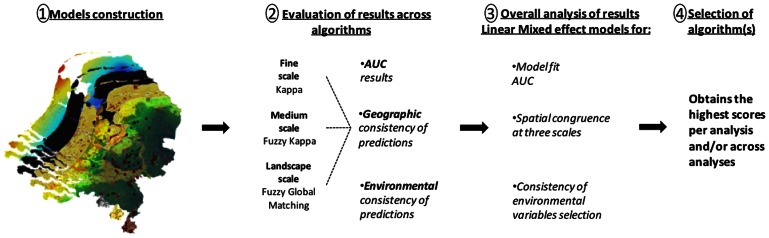
SDM’s analysis framework. Framework for analysing the algorithms adequacy for modelling our species distribution by means of model fit, binary predictions similarity and selection of variables importance. These results are analysed across algorithms by means of Linear Mixed Effects models (LME), which will aid in the selection of the most suitable algorithm for modelling our species distributions.

While species distribution modelling is commonly used to inform and guide conservation actions, until now no extensive evaluation of the quality of the many available methods was available [2], [28]. While current species distribution modelling studies commonly select modelling algorithm haphazardly, mainly based on AUC accuracy, our results show that performance is different between algorithms; no single algorithm was performing best for all evaluation metrics (model fit, geographical consistency and environmental niche). We show that a high model fit does not necessarily translate into highly consistent spatial (i.e. consensus approach) or environmental niche predictions, highlighting the need of *a priori* matching of study aims with modelling approach. We designed a modelling workflow (Fig. 4), that one may follow to select the most suitable modelling algorithm(s) and/or approaches for a given aim (e.g. determining the range of spatially restricted species, or identifying algorithms that produce more consistent models for environmental variables selection, given more certainty during analysis of the species’ ecological niche). Such framework is applicable to different species datasets taking into account variation in several important characteristics of species distributions (level of rarity and spatial extent).

## Supporting Information

Figure S1 **Distribution of the records locations of hoverfly species in the Netherlands.** All the localities where hoverflies hove been found are represented by the orange colour. Blue represents the distribution of the locations for the species modelled in this study.
(TIF)

Figure S2 Representation of the environmental space occupied by the modelled species (for the 10 environmental variables used, in different colours representing the species) and the available environmental conditions in the complete study area (graphs in red colour). The selected species cover the vast majority of Netherlands environmental space. The “x” axis represents the range of values for the environmental variable and the “y “axis represents the counts of cells with those conditions. For reference to the variables names and units see Table S3.
(TIF)

Figure S3 **Variation of model fit (i.e. AUC scores) per algorithm per species in the ten repetition runs.** In the graph every number of records corresponds to a species. Values below the dotted line correspond to predictions that are not better than random. See Table 1 and S4 for further details.
(TIF)

Figure S4 **Example of the data overfitting problematic for one of the RF models.** Cells in green represent areas predicted as presences and in grey are the areas predicted as absences, the black dots represent presence records used during the training of the models. The overfitting occurs and the “presences” predictions are mostly constrained to the training records locations.
(TIF)

Figure S5 **Deviance from the average variable contribution per variable and algorithm depending on the number of records.** ***R*** represents the correlation values between these two variables. Only significant correlations are presented. Significance codes: 0 ‘***’ 0.001 ‘**’ 0.01 ‘*’ 0.05.
(TIF)

Figure S6 **Deviance from the average variable contribution per variable depending on the records’ spatial distribution.** ***R*** represents the correlation values between these two variables. Only significant correlations are presented. Significance codes: 0 ‘***’ 0.001 ‘**’ 0.01 ‘*’ 0.05.
(TIF)

Table S1 **Different approaches for producing SDMs are exemplified by the large variety of algorithms used.** In 42 publications from 2012 containing the words “Species Distribution Models” in the title for 2012 (ISI Web of Knowledge, until 26/06/2012) the studies used 19 different algorithms. These studies focus on different aspect of the modelling process (with the “*” symbol).
(DOCX)

Table S2 Description of the species data used for fitting the models.
(DOCX)

Table S3 Environmental variables used for fitting the SDM.
(DOCX)

Table S4 Statistical results of the Linear Mixed Effect models for the AUC values between algorithms and their interaction with the number of records and spatial distribution.
(DOCX)

Table S5 Statistical results of the Linear Mixed Effect models for the maps similarity values at the finer scale (Kappa) between algorithms and their interaction with the number of records and their spatial distribution.
(DOCX)

Table S6 Statistical results of the Linear Mixed Effect models for the maps similarity values at the medium scale (Improved Fuzzy Kappa) between algorithms and their interaction with the number of records and their spatial distribution.
(DOCX)

Table S7 Statistical results of the Linear Mixed Effect models for the maps similarity values at the coarser scale (Fuzzy Global Matching) between algorithms and their interaction with the number of records and their spatial distribution.
(DOCX)

Table S8 Statistical results of the Linear Mixed Effects models for the deviance from the average environmental variable contribution values between algorithms without separating by variable (environmental variable nested in species).
(DOCX)

Table S9 Statistical results of the Linear Mixed Effect models results for the deviance from the average environmental variable contribution values between algorithms for the same variable.
(DOCX)
