# Supplementary figures and images for: Fit-for-Purpose: Species Distribution Model Performance Depends on Evaluation Criteria – Dutch Hoverflies as a Case Study
Source: PLoS One. 2013 May 14;8(5):e63708. doi: 10.1371/journal.pone.0063708 (PMC3653807; doi:10.1371/journal.pone.0063708)

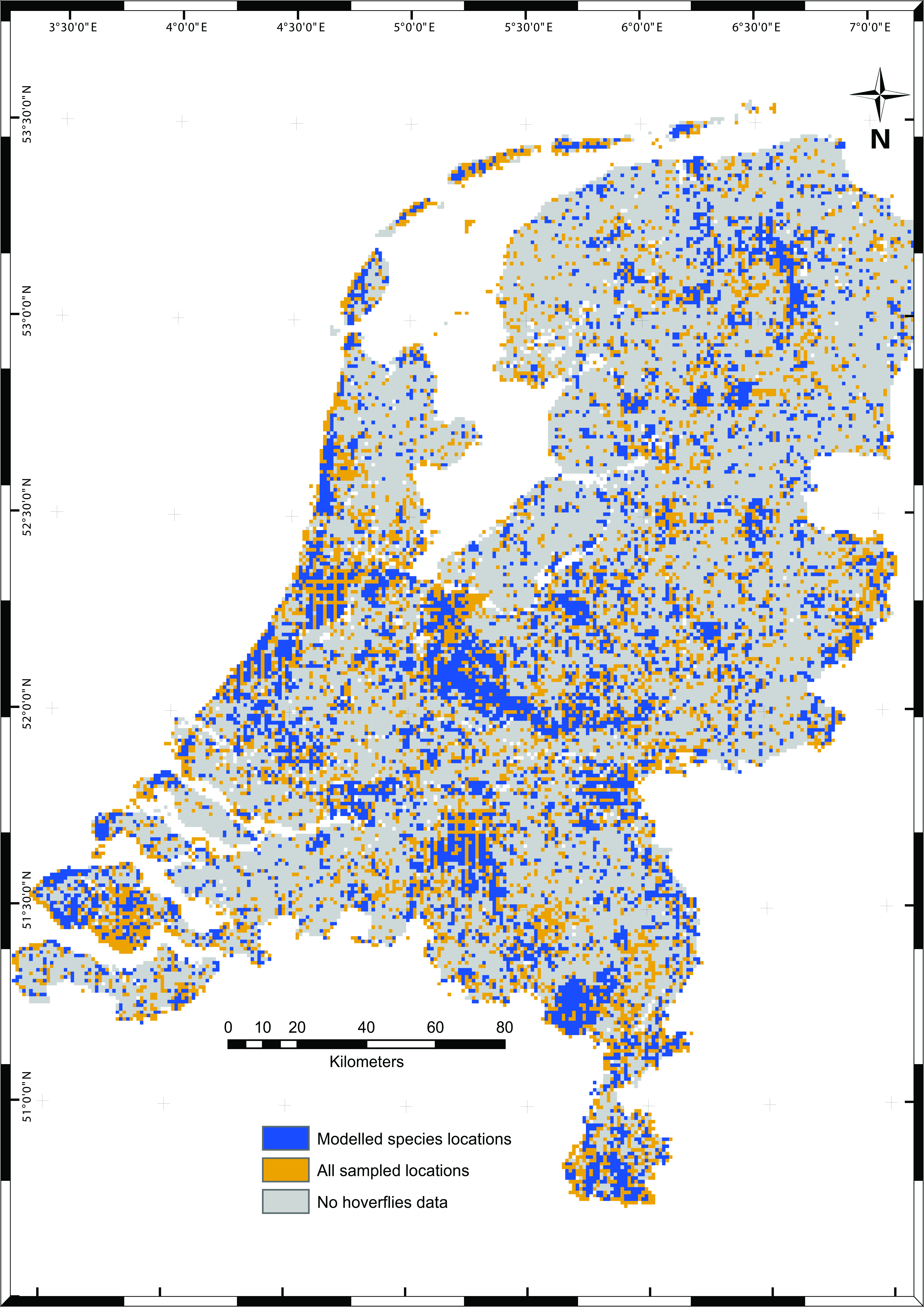

Supplement: Figure S1 — Distribution of the records locations of hoverfly species in the Netherlands. All the localities where hoverflies hove been found are represented by the orange colour. Blue represents the distribution of the locations for the species modelled in this study. (TIF) [file pone.0063708.s001.tif]

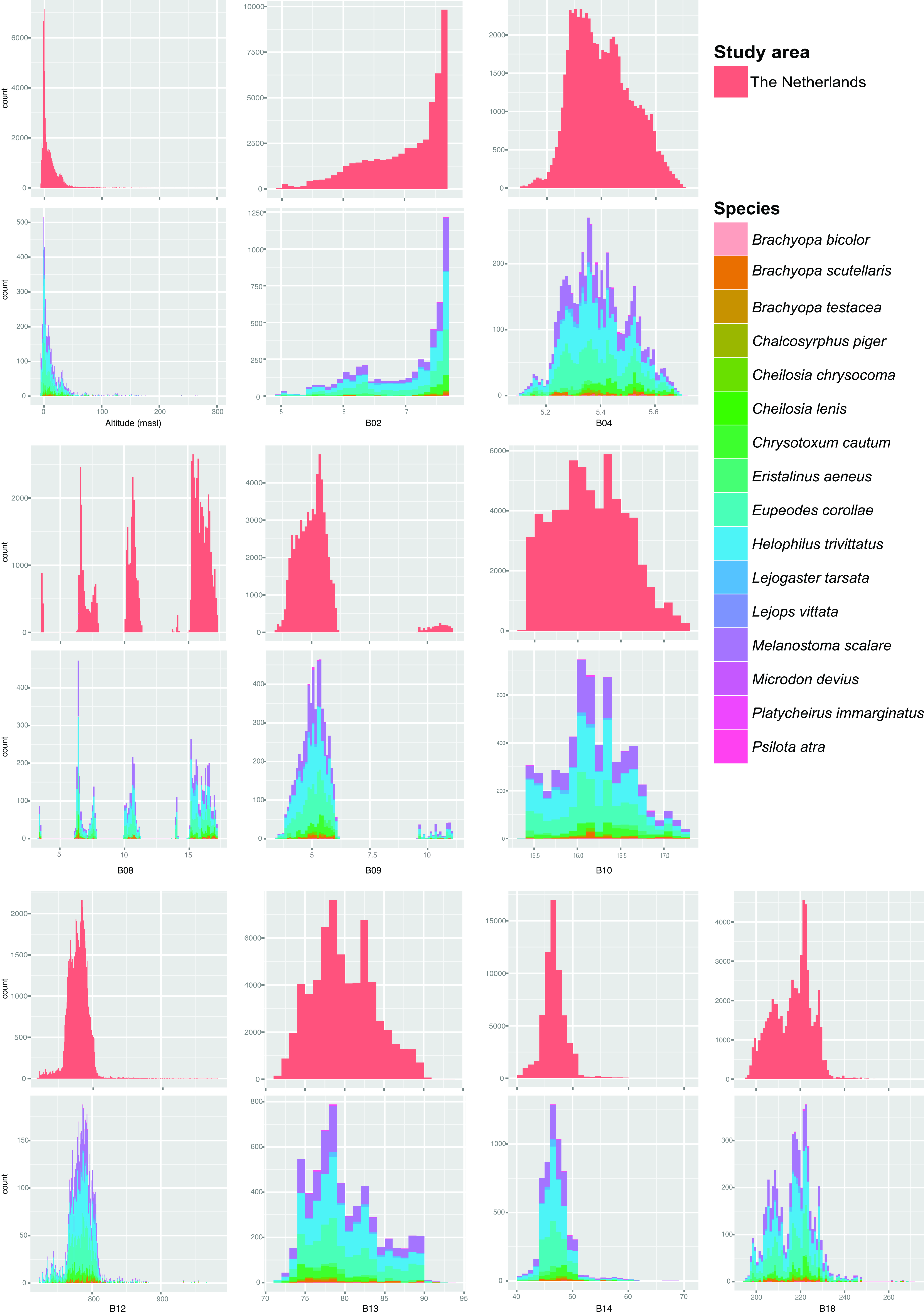

Supplement: Figure S2 — Representation of the environmental space occupied by the modelled species (for the 10 environmental variables used, in different colours representing the species) and the available environmental conditions in the complete study area (graphs in red colour). The selected species cover the vast majority of Netherlands environmental space. The “x” axis represents the range of values for the environmental variable and the “y “axis represents the counts of cells with those conditions. For reference to the variables names and units see Table S3. (TIF) [file pone.0063708.s002.tif]

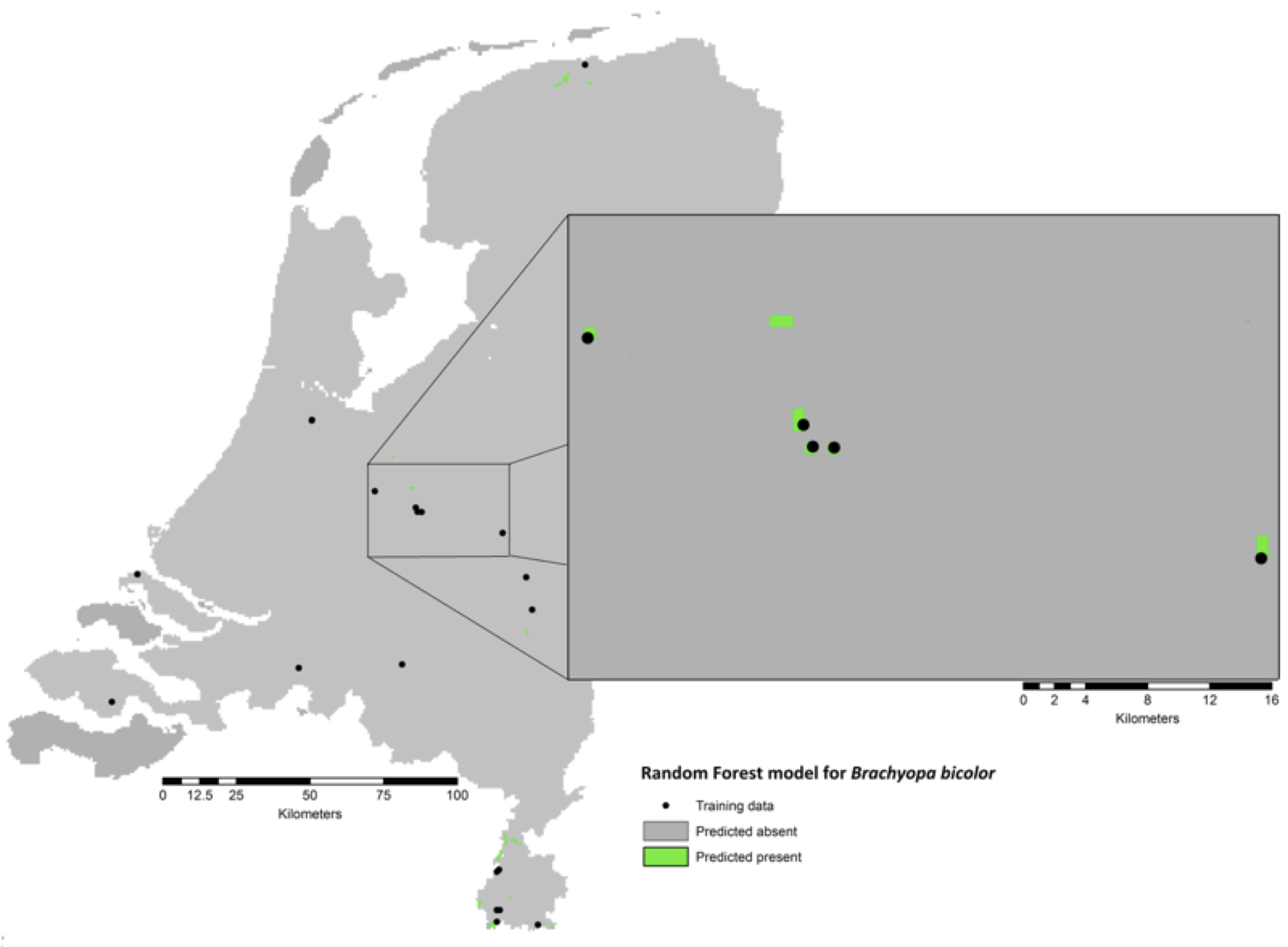

Supplement: Figure S4 — Example of the data overfitting problematic for one of the RF models. Cells in green represent areas predicted as presences and in grey are the areas predicted as absences, the black dots represent presence records used during the training of the models. The overfitting occurs and the “presences” predictions are mostly constrained to the training records locations. (TIF) [file pone.0063708.s004.tif]

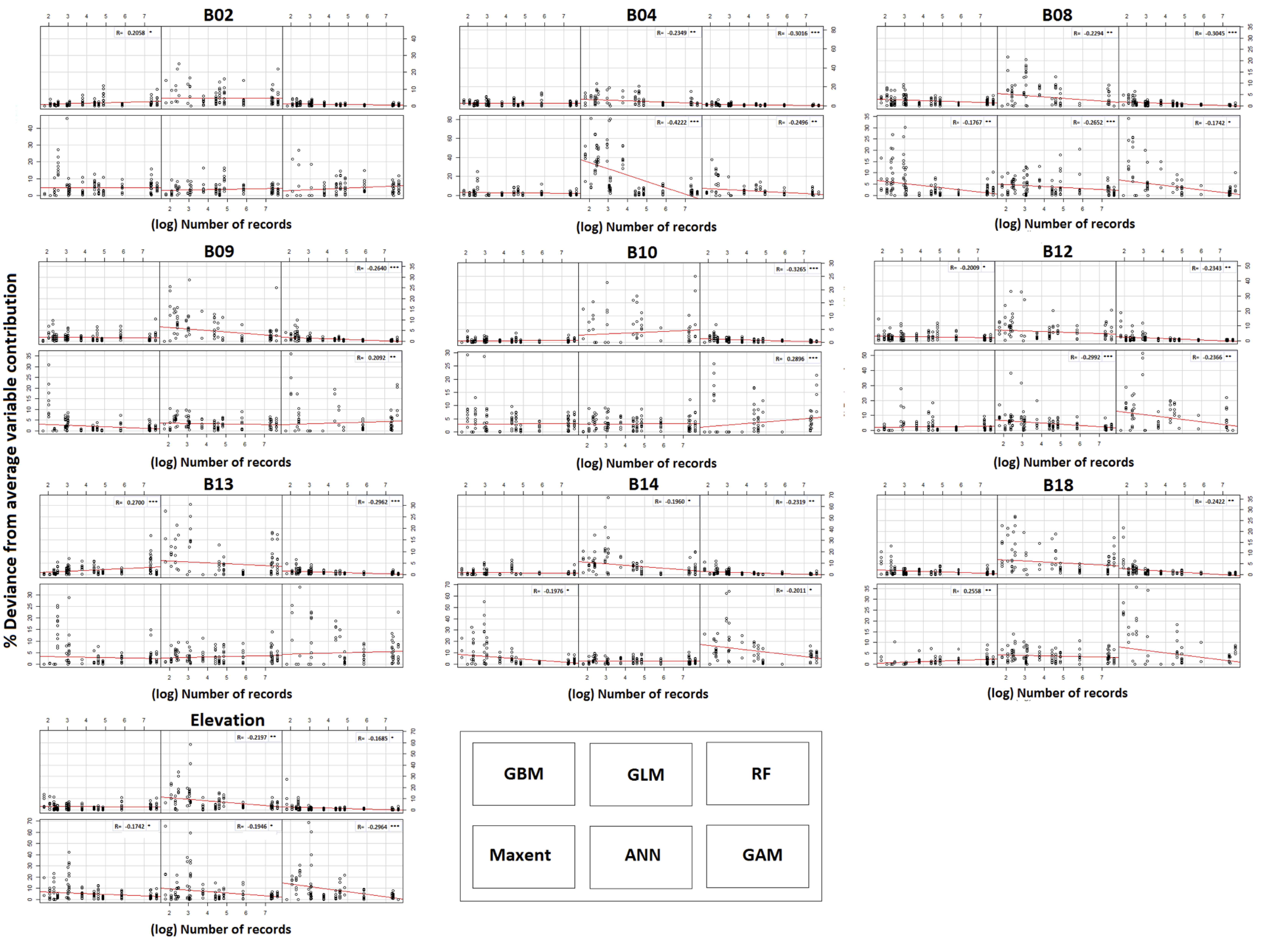

Supplement: Figure S5 — Deviance from the average variable contribution per variable and algorithm depending on the number of records. R represents the correlation values between these two variables. Only significant correlations are presented. Significance codes: 0 ‘***’ 0.001 ‘**’ 0.01 ‘*’ 0.05. (TIF) [file pone.0063708.s005.tif]

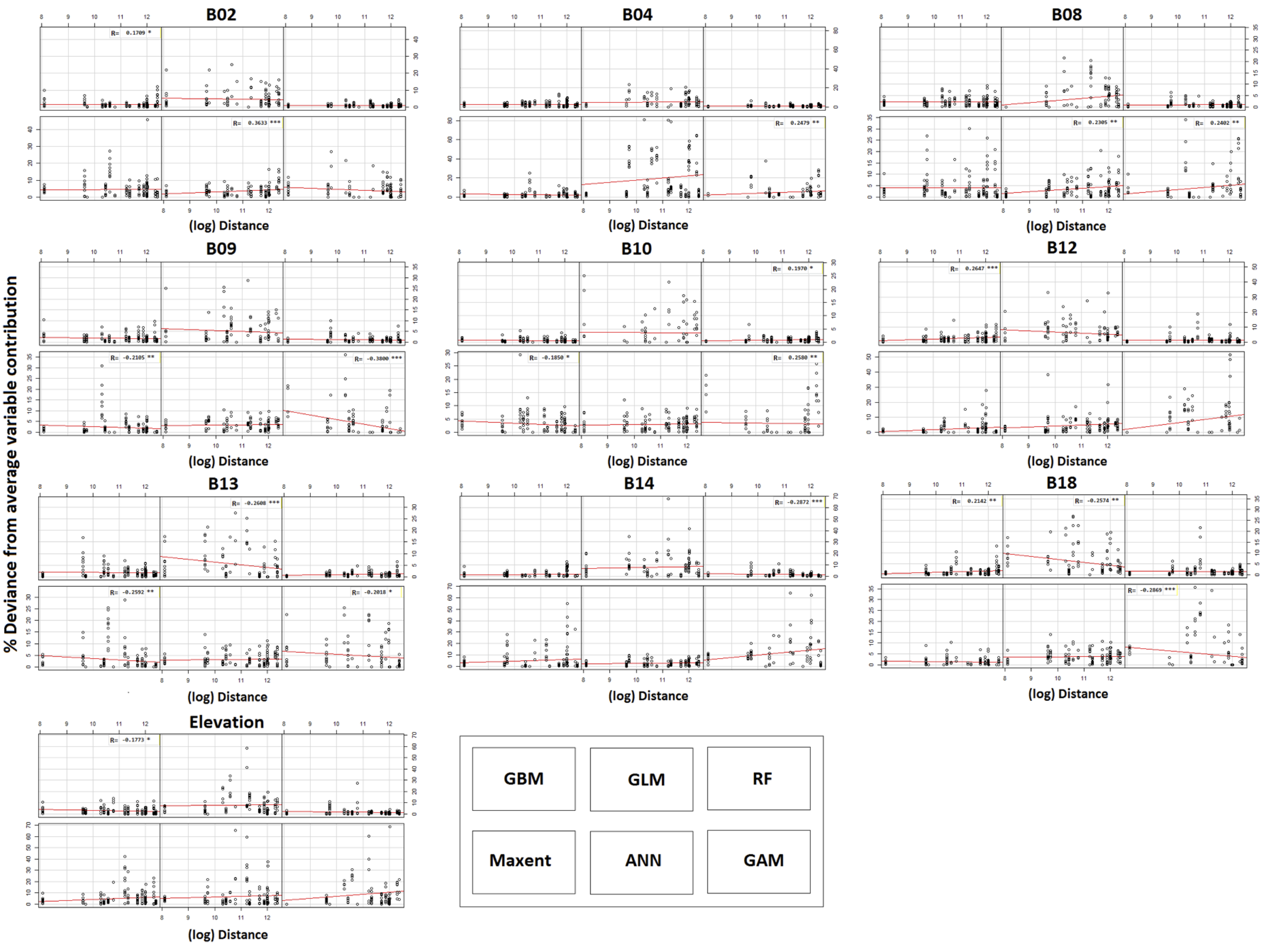

Supplement: Figure S6 — Deviance from the average variable contribution per variable depending on the records’ spatial distribution. R represents the correlation values between these two variables. Only significant correlations are presented. Significance codes: 0 ‘***’ 0.001 ‘**’ 0.01 ‘*’ 0.05. (TIF) [file pone.0063708.s006.tif]
