# Supplementary material for: Fit-for-Purpose: Species Distribution Model Performance Depends on Evaluation Criteria – Dutch Hoverflies as a Case Study
Source: PLoS One. 2013 May 14;8(5):e63708. doi: 10.1371/journal.pone.0063708 (PMC3653807; doi:10.1371/journal.pone.0063708)
Supplement: Table S1 — Different approaches for producing SDMs are exemplified by the large variety of algorithms used. In 42 publications from 2012 containing the words “Species Distribution Models” in the title for 2012 (ISI Web of Knowledge, until 26/06/2012) the studies used 19 different algorithms. These studies focus on different aspect of the modelling process (with the “*” symbol). (DOCX) [file pone.0063708.s007.docx]

**Table S1**. Different approaches for producing SDMs are exemplified by the large variety of algorithms used. In 42 publications from 2012 containing the words “Species Distribution Models” in the title for 2012 (ISI Web of Knowledge, until 26/06/2012) the studies used 19 different algorithms. These studies focus on different aspect of the modelling process (with the “*” symbol).

| Author | Title | Year | Modelling Single species | Modelling Multi species | Effect of number of records | Effect of records distribution | Interested in  Environmental variables | Algorithm(s) used |
| --- | --- | --- | --- | --- | --- | --- | --- | --- |
| Adhikari, D.  Barik, S. K.  Upadhaya, K. | Habitat distribution modelling for reintroduction of Ilex khasiana Purk, a critically endangered tree species of northeastern India | 2012 | ***** | **-** | **-** | **-** | **-** | Maxent |
| Ashcroft, Michael B.  French, Kristine O.  Chisholm, Laurie A. | A simple post-hoc method to add spatial context to predictive species distribution models | 2012 | **-** | ***** | **-** | **-** | **-** | Maxent |
| Babar, Shilpa  Amarnath, Giriraj  Reddy, C. S.  Jentsch, Anke  Sudhakar, S. | Species distribution models: ecological explanation and prediction of an endemic and endangered plant species (Pterocarpus santalinus L.f.) | 2012 | ***** | **-** | **-** | **-** | **-** | GARP  MAXENT  BIOCLIM |
| Barbet-Massin, Morgane  Jiguet, Frederic  Albert, Cecile Helene  Thuiller, Wilfried | Selecting pseudo-absences for species distribution models: how, where and how many? | 2012 | **-** | ***** | ***** | **-** | **-** | GLM  GAM  MARS  MDA  CTA  BRT  RF |
| Bateman, Brooke L.  VanDerWal, Jeremy  Johnson, Christopher N. | Nice weather for bettongs: using weather events, not climate means, in species distribution models | 2012 | **-** | ***** | **-** | **-** | ***** | Maxent |
| Bean, William T.  Stafford, Robert  Brashares, Justin S. | The effects of small sample size and sample bias on threshold selection and accuracy assessment of species distribution models | 2012 | ***** | **-** | ***** | **-** | **-** | Maxent |
| Bentlage, Bastian  Shcheglovitova, Mariya | NichePy: modular tools for estimating the similarity of ecological niche and species distribution models | 2012 | **-** | ***** | **-** | **-** | **-** | RF  ENFA  Ensemble of models |
| Bidinger, K.  Loetters, S.  Roedder, D.  Veith, M. | Species distribution models for the alien invasive Asian Harlequin ladybird (Harmonia axyridis) | 2012 | ***** | **-** | **-** | **-** | ***** | Maxent |
| Bonthoux, Sebastien  Balent, Gerard | Point count duration: five minutes are usually sufficient to model the distribution of bird species and to study the structure of communities for a French landscape | 2012 | **-** | ***** | **-** | **-** | **-** | GLM  GAM  ANN  BRT  MDA  RF  MARS |
| Foltete, Jean-Christophe  Clauzel, Celine  Vuidel, Gilles  Tournant, Pierline | Integrating graph-based connectivity metrics into species distribution models | 2012 | ***** | **-** | **-** | **-** | **-** | GLM |
| Freeman, Elizabeth A.  Moisen, Gretchen G.  Frescino, Tracey S. | Evaluating effectiveness of down-sampling for stratified designs and unbalanced prevalence in Random Forest models of tree species distributions in Nevada | 2012 | **-** | **-** | ***** | **-** | **-** | RF |
| Gomez-Rodriguez, Carola  Bustamante, Javier  Diaz-Paniagua, Carmen  Guisan, Antoine | Integrating detection probabilities in species distribution models of amphibians breeding in Mediterranean temporary ponds | 2012 | **-** | ***** | **-** | **-** | ***** | GLM |
| Hanberry, B. B.  He, H. S.  Dey, D. C. | Sample sizes and model comparison metrics for species distribution models | 2012 | **-** | ***** | ***** | **-** | **-** | RF |
| Hassall, Christopher | Predicting the distributions of under-recorded Odonata using species distribution models | 2012 | **-** | ***** | ***** | **-** | ***** | GLM |
| Heikkinen, Risto K.  Marmion, Mathieu  Luoto, Miska | Does the interpolation accuracy of species distribution models come at the expense of transferability? | 2012 | **-** | ***** | **-** | **-** | **-** | GAM  MARS  GLM  MAXENT  ANN  GBM  RF  GARP  CTA  MDA |
| Heinanen, Stefan  Erola, Johnny  von Numers, Mikael | High resolution species distribution models of two nesting water bird species: a study of transferability and predictive performance | 2012 | **-** | ***** | **-** | **-** | ***** | Maxent |
| Hijmans, Robert J. | Cross-validation of species distribution models: removing spatial sorting bias and calibration with a null model | 2012 | **-** | ***** | **-** | **-** | **-** | BIOCLIM  MAXENT |
| Illoldi-Rangel, Patricia  Rivaldi, Chissa-Louise  Sissel, Blake  Trout Fryxell, Rebecca  Gordillo-Perez, Guadalupe  Rodriguez-Moreno, Angel  Williamson, Phillip  Montiel-Parra, Griselda  Sanchez-Cordero, Victor  Sarkar, Sahotra | Species distribution models and ecological suitability analysis for potential tick vectors of lyme disease in Mexico. | 2012 | **-** | ***** | **-** | **-** | **-** | Maxent |
| Jimenez-Valverde, Alberto | \| Insights into the area under the receiver operating characteristic curve (AUC) as a discrimination measure in species distribution modelling \| \| --- \| \|  \| | 2012 | ***** | **-** | **-** | **-** | **-** | GLM |
| Kamino, Luciana H. Y.  Stehmann, Joao Renato  Amaral, Silvana  De Marco, Paulo, Jr.  Rangel, Thiago F.  de Siqueira, Marinez F.  De Giovanni, Renato  Hortal, Joaquin | Challenges and perspectives for species distribution modelling in the neotropics | 2012 | **-** | ***** | ***** | **-** | ***** | BIOCLIM  GAM  GARP  MARS  EED  ANN |
| Loe, Leif Egil  Bonenfant, Christophe  Meisingset, Erling L.  Mysterud, Atle | Effects of spatial scale and sample size in GPS-based species distribution models: are the best models trivial for red deer management? | 2012 | ***** | **-** | ***** | **-** | **-** | GLM |
| Loehle, Craig | Relative frequency function models for species distribution modeling | 2012 | **-** | ***** | **-** | **-** | **-** | RFF |
| Lundy, Mathieu G.  Buckley, Daniel J.  Boston, Emma S. M.  Scott, David D.  Prodoehl, Paulo A.  Marnell, Ferdia  Teeling, Emma C.  Montgomery, W. Ian | Behavioural context of multi-scale species distribution models assessed by radio-tracking | 2012 | ***** | **-** | **-** | **-** | ***** | GLM |
| Machado-Machado, Elia Axinia | Empirical mapping of suitability to dengue fever in Mexico using species distribution modeling | 2012 | ***** | **-** | **-** | **-** | ***** | Maxent |
| Mateo, Ruben G.  Felicisimo, Angel M.  Pottier, Julien  Guisan, Antoine  Munoz, Jesus | Do Stacked Species Distribution Models Reflect Altitudinal Diversity Patterns? | 2012 | **-** | ***** | **-** | **-** | **-** | BRT  GLM  MARS  GARP  GMD  Maxent  Ensemble of models |
| Matin, Shafique  Chitale, Vishwas Sudhir  Behera, Mukunda Dev  Mishra, Birupakshya  Roy, Partha Sarathi | Fauna data integration and species distribution modelling as two major advantages of geoinformatics-based phytobiodiversity study in today's fast changing climate | 2012 | **-** | ***** | **-** | **-** | **-** | Maxent |
| Meynard, Christine N.  Kaplan, David M. | The effect of a gradual response to the environment on species distribution modeling performance | 2012 | ***** | **-** | **-** | **-** | ***** | GLM  BRT |
| Oliver, Tom H.  Gillings, Simon  Girardello, Marco  Rapacciuolo, Giovanni  Brereton, Tom M.  Siriwardena, Gavin M.  Roy, David B.  Pywell, Richard  Fuller, Robert J. | Population density but not stability can be predicted from species distribution models | 2012 | **-** | ***** | **-** | **-** | **-** | GAM  ANN  RF  MAXENT |
| Parra-Quijano, M.  Iriondo, J. M.  Torres, E. | Improving representativeness of genebank collections through species distribution models, gap analysis and ecogeographical maps | 2012 | **-** | ***** | **-** | **-** | **-** | GLM |
| Royle, J. Andrew  Chandler, Richard B.  Yackulic, Charles  Nichols, James D. | Likelihood analysis of species occurrence probability from presence-only data for modelling species distributions | 2012 | **-** | **-** | **-** | **-** | **-** | Maxent |
| Saupe Erin E., Barve Vijay, Myers Corinne E., Soberón Jorge, Barve Narayani,, et al. | Variation in niche and distribution model performance: The need for a priori assessment of key causal factors | 2012 | **-** | ***** | **-** | ***** | ***** | BIOCLIM  DOMAIN  GAM  GARP  Maxent |
| Sindt, Anthony R.  Pierce, Clay L.  Quist, Michael C. | Fish Species of Greatest Conservation Need in Wadeable Iowa Streams: Current Status and Effectiveness of Aquatic Gap Program Distribution Models | 2012 | **-** | ***** | **-** | **-** | **-** | CTA |
| Stanton, Jessica C.  Pearson, Richard G.  Horning, Ned  Ersts, Peter  Akcakaya, H. Resit | Combining static and dynamic variables in species distribution models under climate change | 2012 | **-** | ***** | **-** | **-** | ***** | Maxent |
| Tisseuil, C.  Vrac, M.  Grenouillet, G.  Wade, A. J.  Gevrey, M.  Oberdorff, T.  Grodwohl, J. -B.  Lek, S. | Strengthening the link between climate, hydrological and species distribution modeling to assess the impacts of climate change on freshwater biodiversity | 2012 | **-** | ***** | **-** | **-** | ***** | BRT |
| Torres, Natalia M.  De Marco Junior, Paulo  Santos, Thiago  Silveira, Leandro  de Almeida Jacomo, Anah T.  Diniz-Filho, Jose A. F. | Can species distribution modelling provide estimates of population densities? A case study with jaguars in the Neotropics | 2012 | ***** | **-** | **-** | **-** | **-** | BIOLCIM  GARP  MAXENT  GBM  RF  MD  DOMAIN  CTA  MARS  MDA  ANN |
| Tyberghein, Lennert  Verbruggen, Heroen  Pauly, Klaas  Troupin, Charles  Mineur, Frederic  De Clerck, Olivier | Bio-ORACLE: a global environmental dataset for marine species distribution modelling | 2012 | ***** | **-** | **-** | **-** | ***** | Maxent |
| Vaclavik, Tomas  Kupfer, John A.  Meentemeyer, Ross K. | Accounting for multi-scale spatial autocorrelation improves performance of invasive species distribution modelling (iSDM) | 2012 | ***** | **-** | **-** | **-** | ***** | GLM  Maxent |
| Vaclavik, Tomas  Meentemeyer, Ross K | Equilibrium or not? Modelling potential distribution of invasive species in different stages of invasion | 2012 | ***** | **-** | **-** | **-** | **-** | ENFA |
| Vanhatalo, Jarno  Veneranta, Lari  Hudd, Richard | Species distribution modeling with Gaussian processes: A case study with the youngest stages of sea spawning whitefish (Coregonus lavaretus L. s.l.) larvae | 2012 | ***** | **-** | **-** | **-** | ***** | GLM |
| Vasconcelos, Tiago S.  Rodriguez, Miguel A.  Hawkins, Bradford A. | Species distribution modelling as a macroecological tool: a case study using New World amphibians | 2012 | **-** | ***** | ***** | **-** | **-** | BIOCLIM  OM_GARP  MAXENT |
| Veloz, Samuel D.  Williams, John W.  Blois, Jessica L.  He, Feng  Otto-Bliesner, Bette  Liu, Zhengyu | No-analog climates and shifting realized niches during the late quaternary: implications for 21st-century predictions by species distribution models | 2012 | **-** | ***** | **-** | **-** | ***** | BRT  MARS  MARS-COM  GAM  GLM  Ensemble of models |
| Williams-Tripp, M.  D'Amico, F. J. N.  Page, C.  Bertrand, A.  Nemoz, M.  Brown, J. A. | Modeling Rare Species Distribution at the Edge: The Case for the Vulnerable Endemic Pyrenean Desman in France | 2012 | ***** | **-** | **-** | **-** | ***** | GLM |

*****Focus; **-**Not important. ANN: Artificial Neural Networks; BIOCLIM: Bioclimatic Envelope; BRT: Boosted Regression Trees; CTA: Classification Tree; DOMAIN: Multivariate distance; GMD: Gower’s Metric; EED: Euclidean Environmental Distance; ENFA: Ecological Niche Factor Analysis; Ensemble: Ensemble of models; GAM: Generalized Additive Models; GARP:  Genetic Algorithm for Rule-set Production; GBM: Generalized Boosting Models; GLM: Generalized Linear Models; MARS: Multivariate Adaptive Regression Splines; Maxent: Maximum Entropy; MD: Mahalanobis Distance; MDA: Mixture Discriminant Analysis; RF: Random Forest; RFF: Relative Frequency Function.

**References Table S1:**

Adhikari, D., Barik, S. K., & Upadhaya, K. (2012). Habitat distribution modelling for reintroduction of Ilex khasiana Purk., a critically endangered tree species of northeastern India. *Ecological Engineering*, *40*, 37–43. doi:10.1016/j.ecoleng.2011.12.004

Ashcroft, M. B., French, K. O., & Chisholm, L. a. (2012). A simple post-hoc method to add spatial context to predictive species distribution models. *Ecological Modelling*, *228*, 17–26. doi:10.1016/j.ecolmodel.2011.12.020

Babar, S., Amarnath, G., Reddy, C. S., Jentsch, A., & Sudhakar, S. (n.d.). Species distribution models : ecological explanation and prediction of an endemic and endangered plant species ( Pterocarpus santalinus L . f .), 1157–1165.

Barbet-Massin, M., Jiguet, F., Albert, C. H., & Thuiller, W. (2012). Selecting pseudo-absences for species distribution models: how, where and how many? *Methods in Ecology and Evolution*, *3*(2), 327–338.

Bateman, B. L., VanDerWal, J., & Johnson, C. N. (2011). Nice weather for bettongs: using weather events, not climate means, in species distribution models. *Ecography*, (June 2011), no–no. doi:10.1111/j.1600-0587.2011.06871.x

Bean, W. T., Stafford, R., & Brashares, J. S. (2012). The effects of small sample size and sample bias on threshold selection and accuracy assessment of species distribution models. *Ecography*, *35*(3), 250–258. doi:10.1111/j.1600-0587.2011.06545.x

Bentlage, B., & Shcheglovitova, M. (2012). NichePy: modular tools for estimating the similarity of ecological niche and species distribution models. *Methods in Ecology and Evolution*, (2011), no–no. doi:10.1111/j.2041-210X.2011.00184.x

Bidinger, K., Lötters, S., Rödder, D., & Veith, M. (2012). Species distribution models for the alien invasive Asian Harlequin ladybird (Harmonia axyridis). *Journal of Applied Entomology*, *136*(1-2), 109–123.

Bonthoux, S., & Balent, G. (2011). Point count duration: five minutes are usually sufficient to model the distribution of bird species and to study the structure of communities for a French landscape. *Journal of Ornithology*, 491–504. doi:10.1007/s10336-011-0766-2

Foltête, J.-C., Clauzel, C., Vuidel, G., & Tournant, P. (2012). Integrating graph-based connectivity metrics into species distribution models. *Landscape Ecology*, 557–569. doi:10.1007/s10980-012-9709-4

Freeman, E. a., Moisen, G. G., & Frescino, T. S. (2012). Evaluating effectiveness of down-sampling for stratified designs and unbalanced prevalence in Random Forest models of tree species distributions in Nevada. *Ecological Modelling*, *233*, 1–10. doi:10.1016/j.ecolmodel.2012.03.007

Gómez-Rodríguez, C., Bustamante, J., Díaz-Paniagua, C., & Guisan, A. (2012). Integrating detection probabilities in species distribution models of amphibians breeding in Mediterranean temporary ponds. *Diversity and Distributions*, *18*(3), 260–272. doi:10.1111/j.1472-4642.2011.00837.x

Hanberry, B. B., He, H. S., & Dey, D. C. (2012). Sample sizes and model comparison metrics for species distribution models. *Ecological Modelling*, *227*, 29–33. doi:10.1016/j.ecolmodel.2011.12.001

Hassall, C. (2011). Predicting the distributions of under-recorded Odonata using species distribution models. *Insect Conservation and Diversity*, no–no. doi:10.1111/j.1752-4598.2011.00150.x

Heikkinen, R. K., Marmion, M., & Luoto, M. (2012). Does the interpolation accuracy of species distribution models come at the expense of transferability? *Ecography*, *35*(3), 276–288. doi:10.1111/j.1600-0587.2011.06999.x

Heinänen, S., Erola, J., & Numers, M. (2012). High resolution species distribution models of two nesting water bird species: a study of transferability and predictive performance. *Landscape Ecology*, *27*(4), 545–555. doi:10.1007/s10980-012-9705-8

Hijmans, R. J. (2012). Cross-validation of species distribution models: removing spatial sorting bias and calibration with a null model. *Ecology*, *93*(3), 679–88.

Illoldi-Rangel, P., Rivaldi, C.-L., Sissel, B., Trout Fryxell, R., Gordillo-Pérez, G., Rodríguez-Moreno, A., Williamson, P., et al. (2012). Species distribution models and ecological suitability analysis for potential tick vectors of lyme disease in Mexico. *Journal of tropical medicine*, *2012*, 959101. doi:10.1155/2012/959101

Jiménez-Valverde, A. (2012). Insights into the area under the receiver operating characteristic curve (AUC) as a discrimination measure in species distribution modelling. *Global Ecology and Biogeography*, *21*, 498–507. doi:10.1111/j.1466-8238.2011.00683.x

Kamino, L. H. Y., Stehmann, J. R., Amaral, S., De Marco, P., Rangel, T. F., De Siqueira, M. F., De Giovanni, R., et al. (2012). Challenges and perspectives for species distribution modelling in the neotropics. *Biology letters*, *8*(3), 324–6. doi:10.1098/rsbl.2011.0942

Loe, L. E., Bonenfant, C., Meisingset, E. L., & Mysterud, A. (2012). Effects of spatial scale and sample size in GPS-based species distribution models: are the best models trivial for red deer management? *European Journal of Wildlife Research*, *58*(1), 195–203. doi:10.1007/s10344-011-0563-5

Loehle, C. (2012). Relative frequency function models for species distribution modeling. *Ecography*, (January), no–no. doi:10.1111/j.1600-0587.2012.07389.x

Lundy, M. G., Buckley, D. J., Boston, E. S. M., Scott, D. D., Prodöhl, P. a., Marnell, F., Teeling, E. C., et al. (2012). Behavioural context of multi-scale species distribution models assessed by radio-tracking. *Basic and Applied Ecology*, *13*(2), 188–195. doi:10.1016/j.baae.2011.12.003

Machado-Machado, E. A. (2012). Empirical mapping of suitability to dengue fever in Mexico using species distribution modeling. *Applied Geography*, *33*, 82–93. doi:10.1016/j.apgeog.2011.06.011

Mateo, R. G., Felicísimo, Á. M., Pottier, J., Guisan, A., & Muñoz, J. (2012). Do Stacked Species Distribution Models Reflect Altitudinal Diversity Patterns? (A. M. Noor, Ed.)*PLoS ONE*, *7*(3), e32586. doi:10.1371/journal.pone.0032586

Matin, S., Chitale, V. S., Behera, M. D., Mishra, B., & Roy, P. S. (2012). Fauna data integration and species distribution modelling as two major advantages of geoinformatics-based phytobiodiversity study in today’s fast changing climate. *Biodiversity and Conservation*, *21*(5), 1229–1250. doi:10.1007/s10531-012-0233-2

Meynard, C. N., & Kaplan, D. M. (2012). The effect of a gradual response to the environment on species distribution modeling performance. *Ecography*, (July 2011), no–no. doi:10.1111/j.1600-0587.2011.07157.x

Oliver, T. H., Gillings, S., Girardello, M., Rapacciuolo, G., Brereton, T. M., Siriwardena, G. M., Roy, D. B., et al. (2012). Population density but not stability can be predicted from species distribution models. *Journal of Applied Ecology*, *49*, 581–590. doi:10.1111/j.1365-2664.2012.02138.x

Parra-Quijano, M., Iriondo, J. M., & Torres, E. (2011). Improving representativeness of genebank collections through species distribution models, gap analysis and ecogeographical maps. *Biodiversity and Conservation*, *21*(1), 79–96. doi:10.1007/s10531-011-0167-0

Royle, J. A., Chandler, R. B., Yackulic, C., & Nichols, J. D. (2012). Likelihood analysis of species occurrence probability from presence-only data for modelling species distributions. *Methods in Ecology and Evolution*, *3*(3), 545–554. doi:10.1111/j.2041-210X.2011.00182.x

Saupe, E. E., Barve, V., Myers, C. E., Soberón, J., Barve, N., Hensz, C. M., Peterson, a. T., et al. (2012). Variation in niche and distribution model performance: The need for a priori assessment of key causal factors. *Ecological Modelling*, *237-238*, 11–22. doi:10.1016/j.ecolmodel.2012.04.001

Sindt, A. R., Pierce, C. L., & Quist, M. C. (2012). Fish Species of Greatest Conservation Need in Wadeable Iowa Streams : Current Status and Effectiveness of Aquatic Gap Program Distribution Models. *North American Journal of Fisheries Management*, *32*(1), 135–146. doi:10.1080/02755947.2012.663456

Stanton, J. C., Pearson, R. G., Horning, N., Ersts, P., & Reşit Akçakaya, H. (2012). Combining static and dynamic variables in species distribution models under climate change. *Methods in Ecology and Evolution*, *3*(2), 349–357. doi:10.1111/j.2041-210X.2011.00157.x

Tisseuil, C., Vrac, M., Grenouillet, G., Wade, a J., Gevrey, M., Oberdorff, T., Grodwohl, J.-B., et al. (2012). Strengthening the link between climate, hydrological and species distribution modeling to assess the impacts of climate change on freshwater biodiversity. *The Science of the total environment*, *424*, 193–201. doi:10.1016/j.scitotenv.2012.02.035

Tyberghein, L., Verbruggen, H., Pauly, K., Troupin, C., Mineur, F., & De Clerck, O. (2012). Bio-ORACLE: a global environmental dataset for marine species distribution modelling. *Global Ecology and Biogeography*, *21*(2), 272–281. doi:10.1111/j.1466-8238.2011.00656.x

Tôrres, N. M., De Marco, P., Santos, T., Silveira, L., De Almeida Jácomo, A. T., & Diniz-Filho, J. a. F. (2012). Can species distribution modelling provide estimates of population densities? A case study with jaguars in the Neotropics. *Diversity and Distributions*, no–no. doi:10.1111/j.1472-4642.2012.00892.x

Vanhatalo, J., Veneranta, L., & Hudd, R. (2012). Species distribution modeling with Gaussian processes: A case study with the youngest stages of sea spawning whitefish (Coregonus lavaretus L. s.l.) larvae. *Ecological Modelling*, *228*, 49–58. doi:10.1016/j.ecolmodel.2011.12.025

Vasconcelos, T. S., Rodríguez, M. Á., & Hawkins, B. a. (2012). Species distribution modelling as a macroecological tool: a case study using New World amphibians. *Ecography*, (July 2011), no–no. doi:10.1111/j.1600-0587.2011.07050.x

Veloz, S. D., Williams, J. W., Blois, J. L., He, F., Otto-Bliesner, B., & Liu, Z. (2012). No-analog climates and shifting realized niches during the late quaternary: implications for 21st-century predictions by species distribution models. *Global Change Biology*, *18*, 1698 – 1713. doi:10.1111/j.1365-2486.2011.02635.x

Václavík, T., Kupfer, J. a., & Meentemeyer, R. K. (2012). Accounting for multi-scale spatial autocorrelation improves performance of invasive species distribution modelling (iSDM). *Journal of Biogeography*, *39*(1), 42–55. doi:10.1111/j.1365-2699.2011.02589.x

Václavík, T., & Meentemeyer, R. K. (2012). Equilibrium or not? Modelling potential distribution of invasive species in different stages of invasion. *Diversity and Distributions*, *18*(1), 73–83. doi:10.1111/j.1472-4642.2011.00854.x

Williams-Tripp, M., D’Amico, F. J. N., Pagé, C., Bertrand, a, Némoz, M., & Brown, J. a. (2012). Modeling rare species distribution at the edge: the case for the vulnerable endemic Pyrenean desman in France. *TheScientificWorldJournal*, *2012*, 612965. doi:10.1100/2012/612965
