# Supplementary material for: Fit-for-Purpose: Species Distribution Model Performance Depends on Evaluation Criteria – Dutch Hoverflies as a Case Study
Source: PLoS One. 2013 May 14;8(5):e63708. doi: 10.1371/journal.pone.0063708 (PMC3653807; doi:10.1371/journal.pone.0063708)
Supplement: Table S2 — Description of the species data used for fitting the models. (DOCX) [file pone.0063708.s008.docx]

**Table S2.** Description of the species data used for fitting the models.

| Species | Number of records | *Distance (Km) |
| --- | --- | --- |
| *Brachyopa bicolor* | 19 | 165.36 |
| *Brachyopa scutellaris* | 81 | 160.65 |
| *Brachyopa testacea* | 22 | 74.95 |
| *Chalcosyrphus piger* | 21 | 83.20 |
| *Cheilosia chrysocoma* | 43 | 162.32 |
| *Cheilosia lenis* | 11 | 16.75 |
| *Chrysotoxum cautum* | 346 | 119.69 |
| *Eristalinus aeneus* | 127 | 238.35 |
| *Eupeodes corollae* | 1578 | 15.12 |
| *Helophilus trivittatus* | 2094 | 3.29 |
| *Lejogaster tarsata* | 100 | 142.41 |
| *Lejops vittata* | 8 | 29.73 |
| *Melanostoma scalare* | 1512 | 33.58 |
| *Microdon devius* | 6 | 48.00 |
| *Platycheirus immarginatus* | 10 | 219.69 |
| *Psilota atra* | 12 | 39.51 |
| *Distance represents the 3^th.^ quartile distance between the most separated record locations for the focus species. More information about the species database can be obtained contacting directly the European Invertebrate Survey – The Netherlands, PO Box 9517, 2300 RA Leiden, The Netherlands; <http://www.eis-nederland.nl>. | | |
