# Supplementary material for: Fit-for-Purpose: Species Distribution Model Performance Depends on Evaluation Criteria – Dutch Hoverflies as a Case Study
Source: PLoS One. 2013 May 14;8(5):e63708. doi: 10.1371/journal.pone.0063708 (PMC3653807; doi:10.1371/journal.pone.0063708)
Supplement: Table S3 — Environmental variables used for fitting the SDM. (DOCX) [file pone.0063708.s009.docx]

**Table S3.** Environmental variables used for fitting the SDM.

| **Name** | **Description** | **Unit** | **Mean** | **Range** | **Std. Dev.** |
| --- | --- | --- | --- | --- | --- |
| B02 | Mean Diurnal Range | °C | 6.3 | 4.9 – 7.8 | 8.6 |
| B04 | Temperature Seasonality | °C | 5.4 | 5 – 5.7 | 0.1 |
| B08 | Mean Temperature of Wettest Quarter | °C | 11.5 | 3.4 – 17.3 | 4.2 |
| B09 | Mean Temperature of Driest Quarter | °C | 6.9 | 3.4 – 11.2 | 2.8 |
| B10 | Mean Temperature of Warmest Quarter | °C | 16.3 | 15.3 - 17.3 | 0.6 |
| B12 | Annual Precipitation | mm | 824.7 | 723 - 983 | 63.9 |
| B13 | Precipitation of Wettest Month | mm | 82.5 | 71 - 94 | 6.9 |
| B14 | Precipitation of Driest Month | mm | 55.5 | 40 - 71 | 9.2 |
| B18 | Precipitation of Warmest Quarter | mm | 231.6 | 194 - 272 | 22.2 |
| Elevation | Elevation range | masl | 109 | -8 - 300 | 72.6 |

The data has a resolution of ≈1 km^2^ (Hijmans et al. 2005, http://www.worldclim.org).
