# Supplementary material for: Fit-for-Purpose: Species Distribution Model Performance Depends on Evaluation Criteria – Dutch Hoverflies as a Case Study
Source: PLoS One. 2013 May 14;8(5):e63708. doi: 10.1371/journal.pone.0063708 (PMC3653807; doi:10.1371/journal.pone.0063708)
Supplement: Table S4 — Statistical results of the Linear Mixed Effect models for the AUC values between algorithms and their interaction with the number of records and spatial distribution. (DOCX) [file pone.0063708.s010.docx]

**Table S4.** Statistical results of the Linear Mixed Effect models for the AUC values between algorithms and their interaction with the number of records and spatial distribution.

| **Algorithms** | **Estimate** | **z value** | **Pr(>\|z\|)** |
| --- | --- | --- | --- |
| Max vs ANN | 1.6890 | 10.6090 | **<0.01** |
| Max vs GAM | 0.3988 | 2.5060 | 0.2087 |
| Max vs GBM | 1.0940 | 6.8710 | **<0.01** |
| Max vs GLM | 0.9014 | 5.6630 | **<0.01** |
| Max vs RF | 1.1460 | 7.1990 | **<0.01** |
| Max vs Con | -0.4769 | -2.9960 | 0.0596 |
| ANN vs GAM | -1.2900 | -8.1040 | **<0.01** |
| ANN vs GBM | -0.5950 | -3.7380 | **<0.01** |
| ANN vs GLM | -0.7873 | -4.9460 | **<0.01** |
| ANN vs RF | -0.5428 | -3.4100 | **0.0165** |
| ANN vs Con | -2.1660 | -13.6050 | **<0.01** |
| GAM vs GBM | 0.6948 | 4.3650 | **<0.01** |
| GAM vs GLM | 0.5026 | 3.1570 | **0.0366** |
| GAM vs RF | 0.7471 | 4.6940 | **<0.01** |
| GAM vs Con | -0.8757 | -5.5020 | **<0.01** |
| GBM vs GLM | -0.1922 | -1.2080 | 0.9555 |
| GBM vs RF | 0.0523 | 0.3280 | 1 |
| GBM vs Con | -1.5710 | -9.8670 | **<0.01** |
| GLM vs RF | 0.2445 | 1.5360 | 0.8299 |
| GLM vs Con | -1.3780 | -8.6590 | **<0.01** |
| RF vs Con | -1.6230 | -10.1950 | **<0.01** |
| Max vs records | -0.0012 | -5.1640 | **<0.01** |
| ANN vs records | -0.0003 | -1.3120 | 0.9267 |
| GAM vs records | -0.0010 | -4.3280 | **<0.01** |
| GBM vs records | -0.0006 | -2.4080 | 0.2579 |
| GLM vs records | -0.0008 | -3.2080 | **0.0317** |
| RF vs records | -0.0006 | -2.4140 | 0.2537 |
| Con vs records | -0.0012 | -4.9600 | **<0.01** |
| Max vs distance | 0.0000 | -2.6830 | 0.1378 |
| ANN vs distance | 0.0000 | -0.2010 | 1 |
| GAM vs distance | 0.0000 | -1.8910 | 0.5998 |
| GBM vs distance | 0.0000 | -0.1700 | 1 |
| GLM vs distance | 0.0000 | -0.7300 | 0.999 |
| RF vs distance | 0.0000 | 0.0260 | 1 |
| Con vs distance | 0.0000 | -2.0140 | 0.51 |

The estimates are the values as obtained in the mixed model without being *logit* back-transformed. The sign of the estimate apply for the first algorithm being compared against the second. The positive sign points to algorithms that render higher values -better fits. Max= Maxent, Con= Consensus approach. Corrected Tukey’s *P values* reported.
