# Supplementary material for: Fit-for-Purpose: Species Distribution Model Performance Depends on Evaluation Criteria – Dutch Hoverflies as a Case Study
Source: PLoS One. 2013 May 14;8(5):e63708. doi: 10.1371/journal.pone.0063708 (PMC3653807; doi:10.1371/journal.pone.0063708)
Supplement: Table S7 — Statistical results of the Linear Mixed Effect models for the maps similarity values at the coarser scale (Fuzzy Global Matching) between algorithms and their interaction with the number of records and their spatial distribution. (DOCX) [file pone.0063708.s013.docx]

**Table S7.** Statistical results of the Linear Mixed Effect models for the maps similarity values at the coarser scale (Fuzzy Global Matching) between algorithms and their interaction with the number of records and their spatial distribution.

| **Algorithms** | **Estimate** | **z value** | **Pr(>\|z\|)** |
| --- | --- | --- | --- |
| Max vs ANN | 1.4940 | 15.9990 | **<0.001** |
| Max vs GAM | -0.1440 | -1.5420 | 0.81365 |
| Max vs GBM | -0.5910 | -6.3300 | **<0.001** |
| Max vs GLM | -0.0231 | -0.2480 | 1 |
| Max vs RF | -0.9389 | -10.0560 | **<0.001** |
| Max vs Con | -0.0857 | -0.9180 | 0.99159 |
| ANN vs GAM | -1.6380 | -17.5410 | **<0.001** |
| ANN vs GBM | -2.0850 | -22.3290 | **<0.001** |
| ANN vs GLM | -1.5170 | -16.2460 | **<0.001** |
| ANN vs RF | -2.4330 | -26.0550 | **<0.001** |
| ANN vs Con | -1.5790 | -16.9170 | **<0.001** |
| GAM vs GBM | -0.4470 | -4.7880 | **<0.001** |
| GAM vs GLM | 0.1209 | 1.2940 | 0.92416 |
| GAM vs RF | -0.7949 | -8.5140 | **<0.001** |
| GAM vs Con | 0.0582 | 0.6240 | 0.99959 |
| GBM vs GLM | 0.5679 | 6.0820 | **<0.001** |
| GBM vs RF | -0.3479 | -3.7260 | **0.00486** |
| GBM vs Con | 0.5053 | 5.4120 | **<0.001** |
| GLM vs RF | -0.9158 | -9.8090 | **<0.001** |
| GLM vs Con | -0.0626 | -0.6710 | 0.99925 |
| RF vs Con | 0.8532 | 9.1380 | **<0.001** |
| Max vs records | -0.0007 | -3.5260 | **0.00996** |
| ANN vs records | -0.0003 | -1.5410 | 0.8148 |
| GAM vs records | -0.0007 | -3.4830 | **0.01229** |
| GBM vs records | -0.0010 | -5.2310 | **<0.001** |
| GLM vs records | -0.0006 | -3.3910 | **0.01626** |
| RF vs records | -0.0010 | -5.5500 | **<0.001** |
| Con vs records | -0.0007 | -3.9620 | **0.00215** |
| Max vs distance | 0.0000 | -2.4560 | 0.22236 |
| ANN vs distance | 0.0000 | 2.4970 | 0.20312 |
| GAM vs distance | 0.0000 | -3.0250 | 0.0523 |
| GBM vs distance | 0.0000 | -2.1470 | 0.40086 |
| GLM vs distance | 0.0000 | -2.6080 | 0.15775 |
| RF vs distance | 0.0000 | -0.4670 | 0.99997 |
| Con vs distance | 0.0000 | -1.8400 | 0.61951 |

The estimates are the values as obtained in the mixed model without being logit back-transformed. The sign of the estimate apply for the first algorithm being compared against the second. The positive sign points to algorithms that render higher values -better fits. Max= Maxent, Con= Consensus approach. Corrected Tukey’s *P values* reported.
