# Supplementary material for: Fit-for-Purpose: Species Distribution Model Performance Depends on Evaluation Criteria – Dutch Hoverflies as a Case Study
Source: PLoS One. 2013 May 14;8(5):e63708. doi: 10.1371/journal.pone.0063708 (PMC3653807; doi:10.1371/journal.pone.0063708)
Supplement: Table S8 — Statistical results of the Linear Mixed Effects models for the deviance from the average environmental variable contribution values between algorithms without separating by variable (environmental variable nested in species). (DOCX) [file pone.0063708.s014.docx]

**Table S8.**  Statistical results of the Linear Mixed Effects models for the deviance from the average environmental variable contribution values between algorithms without separating by variable (environmental variable nested in species).

| **Algorithms** | **Estimate** | **z value** | **Pr(>\|z\|)** |
| --- | --- | --- | --- |
| Max vs ANN | -0.4294 | -16.0850 | **<1e-07** |
| Max vs GAM | -0.2503 | -9.3770 | **<1e-07** |
| Max vs GBM | 0.1603 | 6.0040 | **<1e-07** |
| Max vs GLM | -0.4268 | -15.9870 | **<1e-07** |
| Max vs RF | 0.3840 | 14.3870 | **<1e-07** |
| ANN vs GAM | 0.1791 | 6.7080 | **<1e-07** |
| ANN vs GBM | 0.5897 | 22.0890 | **<1e-07** |
| ANN vs GLM | 0.0026 | 0.0990 | 1 |
| ANN vs RF | 0.8134 | 30.4720 | **<1e-07** |
| GAM vs GBM | 0.4106 | 15.3810 | **<1e-07** |
| GAM vs GLM | -0.1764 | -6.6090 | **<1e-07** |
| GAM vs RF | 0.6344 | 23.7640 | **<1e-07** |
| GBM vs GLM | -0.5870 | -21.9910 | **<1e-07** |
| GBM vs RF | 0.2238 | 8.3830 | **<1e-07** |
| GLM vs RF | 0.8108 | 30.3730 | **<1e-07** |

A significant *P value* points to a significance difference between the deviance values presented by each algorithm. The sign of the estimate apply for the first algorithm being compared against the second. The negative sign point to a more consistent algorithm as it renders lower deviances than the second. The estimates are the values as obtained in the mixed model without being log back-transformed. Max= Maxent. Corrected Tukey’s *P values* reported. See Table S9 for a per variable comparison results between algorithms.
