# Supplementary material for: Fit-for-Purpose: Species Distribution Model Performance Depends on Evaluation Criteria – Dutch Hoverflies as a Case Study
Source: PLoS One. 2013 May 14;8(5):e63708. doi: 10.1371/journal.pone.0063708 (PMC3653807; doi:10.1371/journal.pone.0063708)
Supplement: Table S9 — Statistical results of the Linear Mixed Effect models results for the deviance from the average environmental variable contribution values between algorithms for the same variable. (DOCX) [file pone.0063708.s015.docx]

**Table S9.** Statistical results of the Linear Mixed Effect models results for the deviance from the average environmental variable contribution values between algorithms for the same variable.

| **Algorithm** | **Env. Variable** | **Estimate** | **z value** | **Pr(>\|z\|)** |
| --- | --- | --- | --- | --- |
| ANN-Max | B02 | 0.0308 | 0.3470 | 0.9993 |
| ANN-Max | B04 | 1.4344 | 16.1480 | **<0.001** |
| ANN-Max | B08 | 0.1548 | 1.7430 | 0.5033 |
| ANN-Max | B09 | 0.5731 | 6.4520 | **<0.001** |
| ANN-Max | B10 | 0.1873 | 2.1090 | 0.2825 |
| ANN-Max | B12 | 0.6494 | 7.3110 | **<0.001** |
| ANN-Max | B13 | 0.3506 | 3.9460 | **0.0011** |
| ANN-Max | B14 | -0.1361 | -1.5320 | 0.6435 |
| ANN-Max | B18 | 0.8251 | 9.2880 | **<0.001** |
| ANN-Max | Elevation | 0.2244 | 2.5260 | 0.1166 |
| GAM-ANN | B02 | -0.1993 | -2.2440 | 0.2178 |
| GAM-ANN | B04 | -1.1210 | -12.6190 | **<0.001** |
| GAM-ANN | B08 | -0.2298 | -2.5870 | 0.1006 |
| GAM-ANN | B09 | -0.3535 | -3.9790 | **<0.001** |
| GAM-ANN | B10 | -0.3949 | -4.4460 | **<0.001** |
| GAM-ANN | B12 | -0.0947 | -1.0660 | 0.8950 |
| GAM-ANN | B13 | -0.1174 | -1.3220 | 0.7731 |
| GAM-ANN | B14 | 0.9242 | 10.4040 | **<0.001** |
| GAM-ANN | B18 | -0.2269 | -2.5540 | 0.1090 |
| GAM-ANN | Elevation | 0.0226 | 0.2540 | 0.9999 |
| GAM-Max | B02 | -0.1685 | -1.8970 | 0.4039 |
| GAM-Max | B04 | 0.3135 | 3.5290 | **0.00558** |
| GAM-Max | B08 | -0.0750 | -0.8440 | 0.9593 |
| GAM-Max | B09 | 0.2196 | 2.4720 | 0.1322 |
| GAM-Max | B10 | -0.2076 | -2.3370 | 0.1794 |
| GAM-Max | B12 | 0.5548 | 6.2450 | **<0.001** |
| GAM-Max | B13 | 0.2332 | 2.6250 | 0.09128 |
| GAM-Max | B14 | 0.7881 | 8.8720 | **<0.001** |
| GAM-Max | B18 | 0.5982 | 6.7340 | **<0.001** |
| GAM-Max | Elevation | 0.2469 | 2.7800 | 0.0609 |
| GBM-ANN | B02 | -0.6102 | -6.8700 | **<0.001** |
| GBM-ANN | B04 | -1.2910 | -14.5330 | **<0.001** |
| GBM-ANN | B08 | -0.3318 | -3.7350 | **0.0025** |
| GBM-ANN | B09 | -0.4538 | -5.1090 | **<0.001** |
| GBM-ANN | B10 | -0.7768 | -8.7440 | **<0.001** |
| GBM-ANN | B12 | -0.4163 | -4.6860 | **<0.001** |
| GBM-ANN | B13 | -0.4310 | -4.8520 | **<0.001** |
| GBM-ANN | B14 | -0.4282 | -4.8210 | **<0.001** |
| GBM-ANN | B18 | -0.7059 | -7.9460 | **<0.001** |
| GBM-ANN | Elevation | -0.4516 | -5.0830 | **<0.001** |
| GBM-GAM | B02 | -0.4109 | -4.6260 | **<0.001** |
| GBM-GAM | B04 | -0.1700 | -1.9130 | 0.3939 |
| GBM-GAM | B08 | -0.1020 | -1.1480 | 0.8610 |
| GBM-GAM | B09 | -0.1003 | -1.1290 | 0.8693 |
| GBM-GAM | B10 | -0.3819 | -4.2990 | **<0.001** |
| GBM-GAM | B12 | -0.3216 | -3.6210 | **0.0039** |
| GBM-GAM | B13 | -0.3136 | -3.5300 | **0.0056** |
| GBM-GAM | B14 | -1.3525 | -15.2250 | **<0.001** |
| GBM-GAM | B18 | -0.4790 | -5.3920 | **<0.001** |
| GBM-GAM | Elevation | -0.4741 | -5.3370 | **<0.001** |
| GBM-Max | B02 | -0.5794 | -6.5230 | **<0.001** |
| GBM-Max | B04 | 0.1435 | 1.6150 | 0.5884 |
| GBM-Max | B08 | -0.1770 | -1.9920 | 0.3467 |
| GBM-Max | B09 | 0.1193 | 1.3430 | 0.7608 |
| GBM-Max | B10 | -0.5894 | -6.6360 | **<0.001** |
| GBM-Max | B12 | 0.2331 | 2.6240 | 0.09152 |
| GBM-Max | B13 | -0.0804 | -0.9050 | 0.9453 |
| GBM-Max | B14 | -0.5643 | -6.3530 | **<0.001** |
| GBM-Max | B18 | 0.1192 | 1.3420 | 0.7610 |
| GBM-Max | Elevation | -0.2272 | -2.5580 | 0.1079 |
| GLM-ANN | B02 | 0.0720 | 0.8110 | 0.9657 |
| GLM-ANN | B04 | -0.8617 | -9.7010 | **<0.001** |
| GLM-ANN | B08 | -0.2798 | -3.1500 | **0.0202** |
| GLM-ANN | B09 | 0.1219 | 1.3720 | 0.7439 |
| GLM-ANN | B10 | -0.1539 | -1.7330 | 0.5099 |
| GLM-ANN | B12 | 0.1946 | 2.1910 | 0.2420 |
| GLM-ANN | B13 | 0.0350 | 0.3940 | 0.9988 |
| GLM-ANN | B14 | 0.5900 | 6.6420 | **<0.001** |
| GLM-ANN | B18 | 0.1287 | 1.4490 | 0.6970 |
| GLM-ANN | Elevation | 0.1269 | 1.4290 | 0.7095 |
| GLM-GAM | B02 | 0.2714 | 3.0550 | **0.0273** |
| GLM-GAM | B04 | 0.2593 | 2.9180 | **0.0409** |
| GLM-GAM | B08 | -0.0500 | -0.5630 | 0.9933 |
| GLM-GAM | B09 | 0.4754 | 5.3510 | **<0.001** |
| GLM-GAM | B10 | 0.2410 | 2.7130 | 0.0728 |
| GLM-GAM | B12 | 0.2893 | 3.2560 | **0.0142** |
| GLM-GAM | B13 | 0.1524 | 1.7160 | 0.5212 |
| GLM-GAM | B14 | -0.3342 | -3.7620 | **0.0024** |
| GLM-GAM | B18 | 0.3556 | 4.0030 | **<0.001** |
| GLM-GAM | Elevation | 0.1044 | 1.1750 | 0.8490 |
| GLM-GBM | B02 | 0.6823 | 7.6800 | **<0.001** |
| GLM-GBM | B04 | 0.4292 | 4.8320 | **<0.001** |
| GLM-GBM | B08 | 0.0520 | 0.5850 | 0.9920 |
| GLM-GBM | B09 | 0.5757 | 6.4810 | **<0.001** |
| GLM-GBM | B10 | 0.6229 | 7.0120 | **<0.001** |
| GLM-GBM | B12 | 0.6109 | 6.8770 | **<0.001** |
| GLM-GBM | B13 | 0.4660 | 5.2460 | **<0.001** |
| GLM-GBM | B14 | 1.0183 | 11.4630 | **<0.001** |
| GLM-GBM | B18 | 0.8345 | 9.3950 | **<0.001** |
| GLM-GBM | Elevation | 0.5785 | 6.5120 | **<0.001** |
| GLM-Max | B02 | 0.1028 | 1.1580 | 0.8569 |
| GLM-Max | B04 | 0.5727 | 6.4470 | **<0.001** |
| GLM-Max | B08 | -0.1250 | -1.4070 | 0.7228 |
| GLM-Max | B09 | 0.6950 | 7.8240 | **<0.001** |
| GLM-Max | B10 | 0.0334 | 0.3760 | 0.9990 |
| GLM-Max | B12 | 0.8440 | 9.5010 | **<0.001** |
| GLM-Max | B13 | 0.3856 | 4.3400 | **<0.001** |
| GLM-Max | B14 | 0.4540 | 5.1100 | **<0.001** |
| GLM-Max | B18 | 0.9538 | 10.7370 | **<0.001** |
| GLM-Max | Elevation | 0.3513 | 3.9540 | **0.0010** |
| RF-ANN | B02 | -0.7983 | -8.9860 | **<0.001** |
| RF-ANN | B04 | -1.7411 | -19.6000 | **<0.001** |
| RF-ANN | B08 | -0.7283 | -8.1990 | **<0.001** |
| RF-ANN | B09 | -0.7105 | -7.9980 | **<0.001** |
| RF-ANN | B10 | -0.6778 | -7.6310 | **<0.001** |
| RF-ANN | B12 | -0.8074 | -9.0890 | **<0.001** |
| RF-ANN | B13 | -0.6479 | -7.2930 | **<0.001** |
| RF-ANN | B14 | -0.3920 | -4.4130 | **<0.001** |
| RF-ANN | B18 | -0.7494 | -8.4360 | **<0.001** |
| RF-ANN | Elevation | -0.8816 | -9.9240 | **<0.001** |
| RF-GAM | B02 | -0.5990 | -6.7430 | **<0.001** |
| RF-GAM | B04 | -0.6201 | -6.9800 | **<0.001** |
| RF-GAM | B08 | -0.4985 | -5.6120 | **<0.001** |
| RF-GAM | B09 | -0.3570 | -4.0190 | **<0.001** |
| RF-GAM | B10 | -0.2829 | -3.1850 | **0.0181** |
| RF-GAM | B12 | -0.7128 | -8.0240 | **<0.001** |
| RF-GAM | B13 | -0.5305 | -5.9720 | **<0.001** |
| RF-GAM | B14 | -1.3162 | -14.8170 | **<0.001** |
| RF-GAM | B18 | -0.5226 | -5.8830 | **<0.001** |
| RF-GAM | Elevation | -0.9041 | -10.1780 | **<0.001** |
| RF-GBM | B02 | -0.1880 | -2.1170 | 0.2784 |
| RF-GBM | B04 | -0.4501 | -5.0670 | **<0.001** |
| RF-GBM | B08 | -0.3965 | -4.4630 | **<0.001** |
| RF-GBM | B09 | -0.2567 | -2.8900 | **0.0445** |
| RF-GBM | B10 | 0.0990 | 1.1140 | 0.8759 |
| RF-GBM | B12 | -0.3911 | -4.4030 | **<0.001** |
| RF-GBM | B13 | -0.2169 | -2.4420 | 0.1421 |
| RF-GBM | B14 | 0.0363 | 0.4080 | 0.9986 |
| RF-GBM | B18 | -0.0436 | -0.4910 | 0.9970 |
| RF-GBM | Elevation | -0.4300 | -4.8410 | **<0.001** |
| RF-GLM | B02 | -0.8703 | -9.7970 | **<0.001** |
| RF-GLM | B04 | -0.8793 | -9.8990 | **<0.001** |
| RF-GLM | B08 | -0.4485 | -5.0490 | **<0.001** |
| RF-GLM | B09 | -0.8324 | -9.3700 | **<0.001** |
| RF-GLM | B10 | -0.5239 | -5.8980 | **<0.001** |
| RF-GLM | B12 | -1.0020 | -11.2800 | **<0.001** |
| RF-GLM | B13 | -0.6829 | -7.6870 | **<0.001** |
| RF-GLM | B14 | -0.9820 | -11.0550 | **<0.001** |
| RF-GLM | B18 | -0.8781 | -9.8850 | **<0.001** |
| RF-GLM | Elevation | -1.0085 | -11.3530 | **<0.001** |
| RF-Max | B02 | -0.7675 | -8.6400 | **<0.001** |
| RF-Max | B04 | -0.3066 | -3.4520 | **0.0073** |
| RF-Max | B08 | -0.5735 | -6.4560 | **<0.001** |
| RF-Max | B09 | -0.1374 | -1.5460 | 0.6341 |
| RF-Max | B10 | -0.4905 | -5.5220 | **<0.001** |
| RF-Max | B12 | -0.1580 | -1.7790 | 0.4795 |
| RF-Max | B13 | -0.2973 | -3.3470 | **0.0105** |
| RF-Max | B14 | -0.5281 | -5.9450 | **<0.001** |
| RF-Max | B18 | 0.0757 | 0.8520 | 0.9580 |
| RF-Max | Elevation | -0.6572 | -7.3980 | **<0.001** |

A significant *P value* points to a significance difference between the deviance values presented by each algorithm. The sign of the estimate apply for the first algorithm being compared against the second. The negative sign point to a more consistent algorithm as it renders lower deviances than the second. The estimates are the values as obtained in the mixed model without being log back-transformed. Max= Maxent. Corrected Tukey’s *P values* reported. For questions about specific models and/or algorithms formula please contact the author for correspondence.
